# Regulation of alternative splicing in *Drosophila* by 56 RNA binding proteins

**DOI:** 10.1101/gr.192518.115

**Published:** 2015-11

**Authors:** Angela N. Brooks, Michael O. Duff, Gemma May, Li Yang, Mohan Bolisetty, Jane Landolin, Ken Wan, Jeremy Sandler, Benjamin W. Booth, Susan E. Celniker, Brenton R. Graveley, Steven E. Brenner

**Affiliations:** 1 Department of Molecular and Cell Biology, University of California, Berkeley, California 94720, USA;; 2 Broad Institute, Cambridge, Massachusetts 02142, USA;; 3 Department of Medical Oncology, Dana-Farber Cancer Institute, Boston, Massachusetts 02215, USA;; 4 Department of Genetics and Genome Sciences, Institute for Systems Genomics, University of Connecticut Health Center, Farmington, Connecticut 06030, USA;; 5 Department of Genome Dynamics, Lawrence Berkeley National Laboratory, Berkeley, California 94720, USA;; 6 Department of Plant and Microbial Biology, University of California, Berkeley, California 94720, USA

## Abstract

Alternative splicing is regulated by RNA binding proteins (RBPs) that recognize pre-mRNA sequence elements and activate or repress adjacent exons. Here, we used RNA interference and RNA-seq to identify splicing events regulated by 56 *Drosophila* proteins, some previously unknown to regulate splicing. Nearly all proteins affected alternative first exons, suggesting that RBPs play important roles in first exon choice. Half of the splicing events were regulated by multiple proteins, demonstrating extensive combinatorial regulation. We observed that SR and hnRNP proteins tend to act coordinately with each other, not antagonistically. We also identified a cross-regulatory network where splicing regulators affected the splicing of pre-mRNAs encoding other splicing regulators. This large-scale study substantially enhances our understanding of recent models of splicing regulation and provides a resource of thousands of exons that are regulated by 56 diverse RBPs.

Most metazoan genes contain introns that are removed from their primary transcripts (pre-mRNA) by the spliceosome, a macromolecular machine composed of hundreds of proteins and five small RNAs, which joins flanking exons together to generate a mature mRNA. Pre-mRNAs are alternatively spliced when the spliceosome uses different splice sites, thus creating different mRNAs, and frequently proteins, from a single gene. Alternative splicing is an important aspect of gene regulation that is used to produce different transcript isoforms in a tissue-specific (Pan et al. 2008; Wang et al. 2008; Barbosa-Morais et al. 2012; Merkin et al. 2012; Brown et al. 2014) or temporal-specific (Graveley et al. 2011) manner. Alternative splicing is primarily regulated by proteins that bind to the pre-mRNA and act to enhance or inhibit spliceosome assembly, typically at nearby splice sites (Nilsen and Graveley 2010). To date, only about 50 vertebrate RNA binding proteins have been identified as regulators of alternative splicing (Gabut et al. 2008), which is surprising given that there are approximately 100,000 alternative splicing events known in humans (Pan et al. 2008; Wang et al. 2008). To obtain a comprehensive understanding of how splicing is regulated, it is essential to identify splicing regulatory proteins and the specific splicing events they affect.

The best-characterized splicing regulatory proteins are members of the SR and hnRNP protein families (Chen and Manley 2009). These proteins play key roles in both constitutive and alternative splicing and act by several different mechanisms. SR proteins have been shown to help recruit the spliceosome to splice sites and to promote exon inclusion (Chen and Manley 2009), while hnRNPs are thought to primarily repress exon inclusion by antagonizing SR proteins, by directly inhibiting the spliceosome, or by a “looping out” mechanism of regulation (Chen and Manley 2009). Several studies have identified targets of *Drosophila* SR and hnRNP proteins and found very few overlapping effects between the two types of proteins (Blanchette et al. 2005, 2009). A recent study of *Drosophila* SR proteins found that most regulatory targets of SR proteins were coregulated by another SR protein and that they coordinately promoted exon inclusion and skipping (Bradley et al. 2015). A study of hnRNP proteins in human kidney cells identified significant overlaps in the targets of hnRNPs (Huelga et al. 2012). This study also found examples of cross-regulation where one hnRNP can regulate the splicing of pre-mRNAs encoding other hnRNPs (Huelga et al. 2012).

In addition to SR and hnRNP proteins, other RNA binding proteins, including RBFOX2, NOVA1/2, and PTBP1 among others (Ule et al. 2006; Boutz et al. 2007; Yeo et al. 2009), have been shown to regulate alternative splicing in a tissue-regulated or developmentally regulated manner. These proteins, like SR and hnRNP proteins, contain one or more RRM or KH domains that typically interact with RNA in a sequence-specific manner (Perez-Canadillas and Varani 2001).

Unexpectedly, proteins that are core components of the spliceosome have been shown to modulate splicing in yeast and *Drosophila* in a substrate-specific manner (Park et al. 2004; Pleiss et al. 2007). In the *Drosophila* study (Park et al. 2004), the impact of core spliceosomal components was examined on only a few alternative splicing events. Thus, the genome-wide impacts of perturbing core components of the spliceosome in *Drosophila* remain to be determined.

Another set of proteins that appears to play an unexpected role in alternative splicing is the components of the exon junction complex (EJC). The EJC proteins EIF4A3, TSU/RBM8A, and MAGO have been shown to regulate splicing (Ashton-Beaucage et al. 2010; Roignant and Treisman 2010; Michelle et al. 2012). EJC components are deposited near the exon junction after splicing, where they assist in transport, localization, and translation of the processed mRNA (Tange et al. 2004; Isken and Maquat 2008). Additionally, EJC proteins are important in facilitating nonsense-mediated decay (NMD) of transcripts containing a premature stop codon (Tange et al. 2004; Chang et al. 2007).

To gain a more comprehensive understanding of the regulation of alternative splicing, we used RNA interference (RNAi) to deplete 56 proteins, including known splicing regulators such as SR and hnRNP proteins, or putative splicing regulators that contain RNA binding domains, core spliceosomal proteins, and EJC components in *Drosophila melanogaster* S2-DRSC tissue culture cells, and identified transcriptome-wide changes by RNA sequencing (RNA-seq). We identified thousands of targets for these proteins, providing the most extensive genome-wide characterization of targets of splicing regulators in any organism to date.

## Results

### Identification of transcriptome-wide regulatory targets of 56 proteins

To obtain a global understanding of splicing regulation in *D. melanogaster*, we performed RNA-seq of poly(A)+ RNA isolated from S2-DRSC cells that were individually depleted of 56 candidate splicing regulatory proteins. We selected the proteins based on their previously known roles in regulating alternative splicing, their homology to known regulators (Mount and Salz 2000; Park et al. 2004; Barbosa-Morais et al. 2006), or their potential to function as a splicing regulator due to the presence of one or more RRM or KH domains. Each protein was categorized as an SR protein, as hnRNP protein, as a core component of the spliceosome, as part of the EJC, as having evidence of splicing regulation, or as having no prior evidence of splicing regulation (Supplemental Table 1), though these categories are not mutually exclusive.

After confirming depletion of the targeted mRNA by RT-PCR (Supplemental Fig. 1), we prepared poly(A)+ RNA-seq libraries from two biological replicates of each RNAi-depleted sample, as well as from untreated cells, and sequenced each library to generate a total of 18–59 million uniquely aligned, single-end, 75- to 76-bp reads per sample (Supplemental Table 1). All reads were trimmed to 75 bp and aligned to the genome and a set of annotated and novel splice junctions (see Supplemental Methods) using Bowtie (Langmead et al. 2009). To obtain a confident set of novel junctions to use in subsequent analyses, we scored alignments to novel splice junctions using a Shannon entropy measure (Graveley et al. 2011). We used Cufflinks (Trapnell et al. 2010) to quantitate gene and transcript levels in each sample and used JuncBASE (Brooks et al. 2011) to identify and quantitate alternative splicing events.

**Figure 1.**
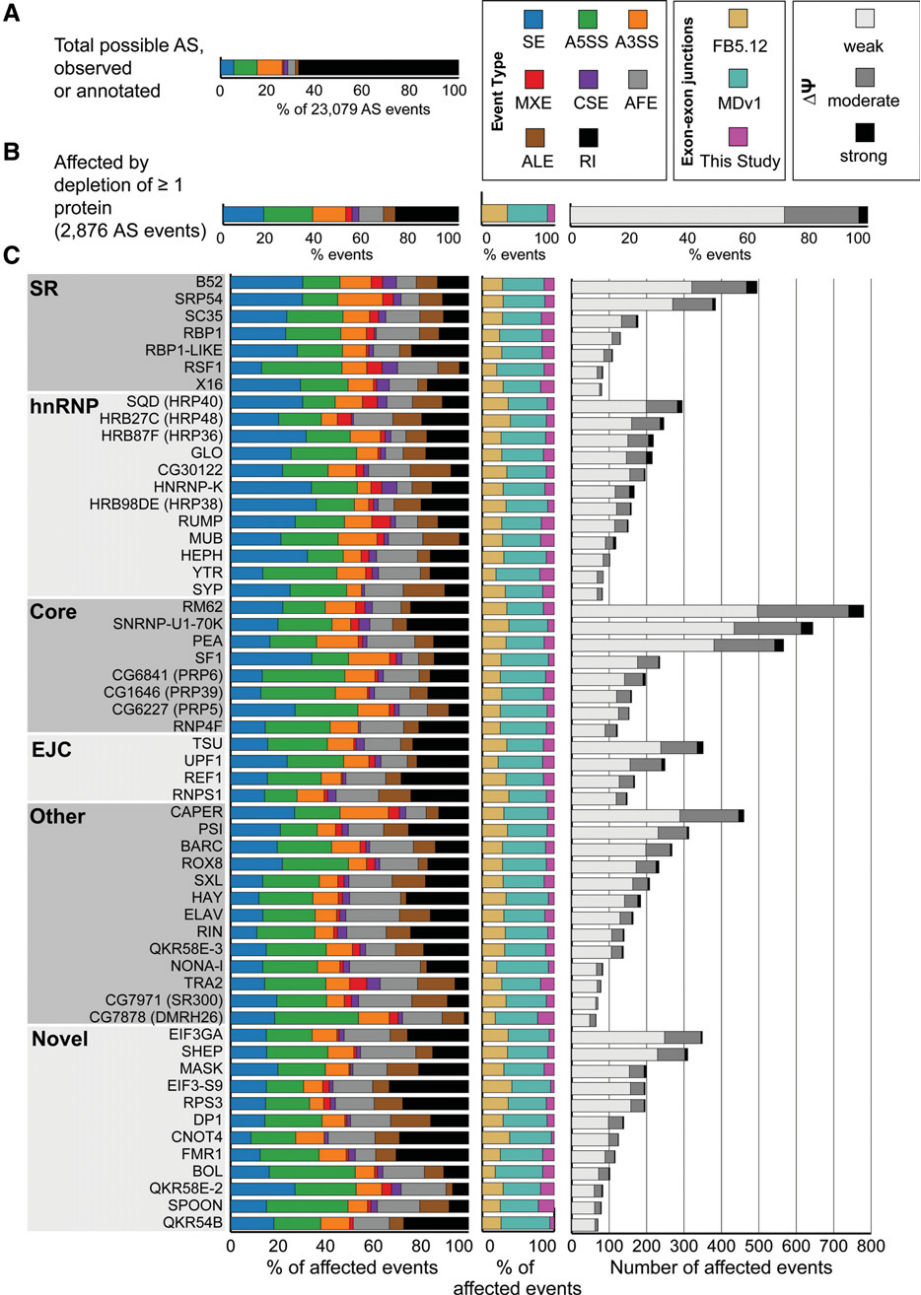
Alternative splicing (AS) events affected by depletion of 56 RNA binding proteins. (*A*) The proportion of each type of AS event that could potentially be regulated by each protein. (*B*) The observed proportion of each type of splicing event affected by depletion of at least one protein is shown alongside the type of exon–exon junctions involved in the splicing event. (*C*) The number, magnitude, type of AS, and type of exon–exon junction affected by each individual knockdown is shown. Each protein is categorized as SR, hnRNP, core splicing factor, exon junction complex (EJC), other known, or novel splicing regulator protein. The legend in the *top right* indicates the color codes used for the splicing event types. (SE) skipped/cassette exon, (A5SS) alternative 5′ splice site, (A3SS) alternative 3′ splice site, (MXE) mutually exclusive exons, (CSE) coordinate skipped/cassette exon, (AFE) alternative first exon, (ALE) alternative last exon, (RI) retained intron. In addition, the legend indicates whether the exon–exon junctions were found in FlyBase 5.12, MDv1 (Graveley et al. 2011), or novel to this study, as well as whether the magnitude of splicing changes observed were weak, moderate, or strong.

We identified 23,079 alternative splicing events that were expressed in our data set (Fig. 1A), 2876 of which were significantly altered by depletion of at least one splicing regulatory protein (Fig. 1B). JuncBASE incorporates unannotated splice junctions when examining splicing alterations. Only 35% of splicing alterations involved solely splice junctions annotated in FlyBase 5.12 (Fig. 1B). Most (55%) splicing changes involved a splice junction reported in a large modENCODE study (MDv1) of the *Drosophila* developmental transcriptome (Fig. 1B), and 10% of events involved splice junctions novel to this study. By using all observed splice junctions in our analysis, we were able to gain a more global picture of splicing changes, which would have been missed if we relied on only annotated events. A splicing event was considered to be altered if the difference in the “percentage spliced in” (PSI, or Ψ) value, the fraction of a gene's mRNAs that contain the exon, in the depleted sample was >10% of a virtual reference and the difference was significant given a false-discovery rate (FDR) of 5% (see Methods). We performed RT-PCR validation experiments for 39 events identified as being significantly regulated in the RNA-seq data and found a significant correlation (*P* < 0.001) between ΔΨ values determined by JuncBASE and by RT-PCR (Supplemental Fig. 2A). Additionally, our set of exons significantly affected by knockdown of *B52*, *Rbp1*, *Rbp1-like*, *Rsf1*, *SC35*, *Srp54*, and *x16* had concordant ΔΨ values with an independent study of these same proteins (Supplemental Fig. 2B; Bradley et al. 2015).

Between 65 and 780 events were affected by depletion of each protein, though each had distinct effects on the number, magnitude, and type of affected splicing events (Fig. 1C). Depletion of core spliceosomal components such as *Rm62*, *snRNP-U1-70K*, and *pea (Prp22)* impacted the largest number of splicing events (over 500 each). Other proteins that had a large impact on splicing include the SR protein B52 and the RNA binding protein CAPER, which both affected over 400 splicing events. The 12 proteins we examined that had no previously known role in alternative splicing affected many splicing events. In fact, depletion of *eIF3ga* and *shep* each affected over 300 events, more than any hnRNP proteins and most SR proteins. EIF3GA is orthologous to human EIF3G, a component of the eIF3 complex involved in translation (Lasko 2000; Hinnebusch 2006). Many splicing regulators have been shown to also affect translation (e.g., Romanelli et al. 2013; Maslon et al. 2014); therefore, other examples of multifunctional RNA binding proteins exist. SHEP is an RNA binding protein that contains two RRM domains, is highly expressed in the nervous system of the fly (Brown et al. 2014), and is involved in gravity sensing (Armstrong et al. 2006). Our results strongly suggest that EIF3GA and SHEP are splicing regulatory proteins; however, additional biochemical studies will be important to further support this function.

Each protein also had distinct effects on the magnitude of individual splicing events. Overall, while the magnitude of some (3%) splicing changes was strong (ΔΨ >50%), most were moderate (ΔΨ 25%–50%) or weak (ΔΨ 10%–25%). The largely weak effects on splicing events upon depletion of the core components of the spliceosome (*Rm62*, *snRNP-U1-70K*, and *pea*) suggest that cells may be fairly insensitive to changes in the levels of spliceosomal components. We confirmed depletion of the targeted mRNAs from the RNA-seq data (Supplemental Fig. 3) and have shown protein depletion for genes with available antibodies in previous studies using the same dsRNA protocols (Park et al. 2004; Olsen et al. 2007). Therefore, we believe the weak splicing effects are not due to inefficient protein depletion. However, as deletions of most genes encoding spliceosomal components are lethal in the fly, it is likely that more complete depletion of these proteins would show larger effects than we observe.

Fifty-two of the proteins examined had a significant preference in the type of alternative splicing event that was affected (χ^2^ test, corrected *P* < 0.05). For example, knockdowns of *snRNP*-*U1-70K*, *CG1646* (*Prp39*), and *Rox8* preferentially affected alternative 5′ splice sites (A5SSs), consistent with their roles as integral U1 snRNP proteins (SNRNP-U1-70K and CG1646 (PRP39)) or U1 snRNP interacting proteins (ROX8) (Fig. 1C). Although the branchpoint recognition protein SF1 does affect alternative 3′ splice site (A3SS) events, cassette exons were most affected by SF1 depletion, consistent with a recent study of human SF1 (Corioni et al. 2011). The observed preferential effects suggest possible mechanisms for proteins without well-defined regulatory mechanisms. For example, *glorund* (*glo*) encodes an hnRNP protein known to regulate translation (Kalifa et al. 2006) and preferentially affects A5SSs and cassette exons, suggesting a role in the regulation 5′ splice site recognition or activation.

### Alternative first exon usage cannot be explained by secondary effects through transcription factors

We were surprised that though they were observed in previous studies of splicing regulators (Brooks et al. 2011; Huelga et al. 2012), a considerable proportion (6%–30%) of alternative splicing events affected by all proteins are alternative first exons (AFEs), as this may involve a change in promoter use (Fig. 1C). To increase our confidence that these were true first exons, we only considered annotated exons (r5.32) (McQuilton et al. 2012) that are also supported by the modENCODE CAGE data sets (Brown et al. 2014). To determine if these observations could be explained by splicing or gene expression changes of transcription factors, we used the modENCODE ChIP-seq data for 41 transcription factors (Negre et al. 2011; Boyle et al. 2014) to predict their downstream target genes based on evidence of strong binding around gene promoters. Although we observed 266 cases where a splicing or gene expression change in a transcription factor co-occurred with an AFE change in their downstream targets, none of the AFE changes occurred consistently with effects on the transcription factor across the samples. Moreover, it is unclear if or how the splicing or gene expression changes observed in the transcription factors impact protein levels or isoforms or whether the interactions observed by ChIP-seq near the promoters of the affected AFEs are functional. Thus, we cannot attribute the observed AFE changes to secondary effects caused by changes in splicing or gene expression of transcription factors. Intriguingly, a recent study (Ji et al. 2013) revealed that SRSF2 associates with the promoter and enhances transcription elongation by recruitment of CDK9 from the 7SK RNA complex. Thus, there are precedents for RNA binding proteins impacting promoter choice, and our results suggest that this may be more widespread than previously recognized. However, we currently do not understand the exact mechanism for our observed changes in widespread AFE changes.

### Gene expression changes are distinct from splicing changes

As many splicing factors not only regulate splicing but also control other RNA processing steps, including transcription, export, and decay (Braunschweig et al. 2013), we examined the impact of depleting each protein on mRNA levels. We identified between 197 and 861 genes in each sample (4194 genes in total) with at least a twofold change in expression in comparison to the untreated sample (*q*-value <0.05) (Supplemental Fig. 4). However, 1670 of the genes that were affected were expressed at low abundance (FPKM < 1) in the untreated sample, indicating that ∼40% of these changes cannot be reliably measured.

Proteins affecting the most splicing events also caused the most changes in gene expression (Supplemental Fig. 5), for example, PEA and RM62. There are, however, exceptions to this trend. The greatest exception is the small ribosomal subunit protein RPS3, which had the second largest effect on mRNA levels (802 genes) but the 23rd largest effect on splicing (188 events). Interestingly, this protein also has been implicated in DNA damage repair via deoxyribophosphodiesterase activity (Wilson et al. 1993). The changes in gene expression may involve a pathway response, instead of a direct effect, of RPS3 on RNA.

Although the relative number of splicing and gene expression changes observed was similar for most proteins, we found no significant overlap of genes with gene expression changes and splicing changes. Thus, the majority of gene expression changes we observed cannot be immediately explained by secondary effects of splicing changes in that gene. Further supporting multifunctional effects, 61% of intronless genes were differentially expressed upon knockdown of at least one RBP, which is more than expected given that 53% of intron-containing genes were differentially expressed (Fisher's exact test, *P* < 0.0001). However, we expect that the full set of splicing regulatory targets would be partially masked by NMD (Kawashima et al. 2014), and some of the differential gene expression may be secondary effects due to changes in transcription or RNA stability. Although additional studies are needed to understand the mechanism of observed gene expression changes, it is clear that looking at both splicing and gene expression changes are important to gain a global picture of transcriptome targets of each RBP.

### Cross-regulation of splicing in transcripts of other regulator proteins

**Figure 2.**
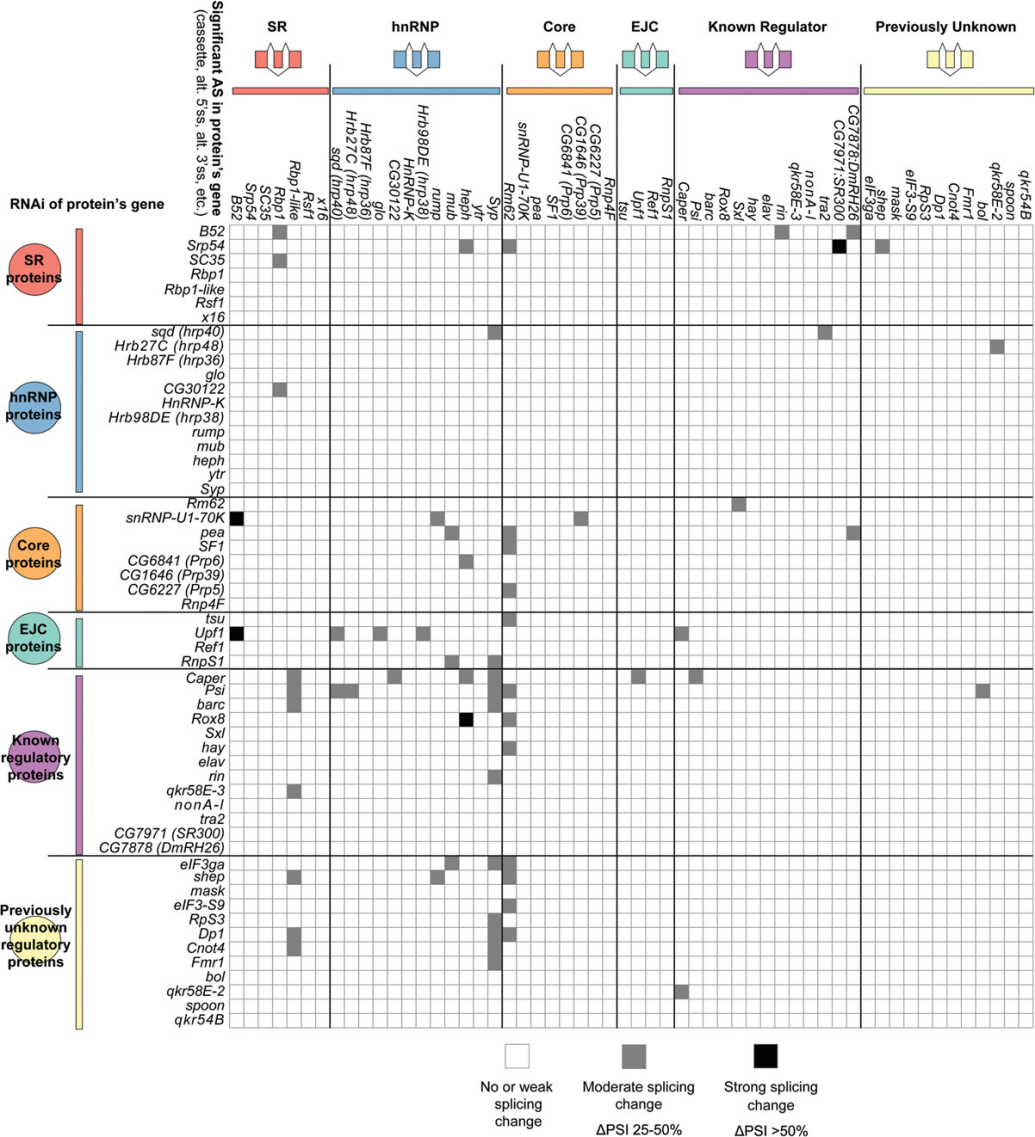
Cross-regulatory splicing networks involving the 56 RNA binding proteins. Based on validation experiments and manual inspection of RNA-seq data, we report observed cross-regulatory effects for moderate to strong splicing changes reported by JuncBASE. The cross-regulation of one protein affecting a splicing event of another protein's pre-mRNA is indicated in the matrix. Although splicing events are diagrammed as cassette exons, effects on all forms of alternative splicing are reported.

Several groups have previously reported that splicing regulators affect the splicing of pre-mRNAs encoding other splicing regulators (Kumar and Lopez 2005; Anko et al. 2012; Huelga et al. 2012). In some of these known cases, the cross-regulation up-regulates expression of an NMD-targeted isoform, thus causing changes in gene expression of the target splicing regulator (Anko et al. 2012; Huelga et al. 2012). In *Drosophila*, a classic example of splicing regulators affecting the function of other splicing regulators is in the sex determination pathway, where SXL regulates splicing of another splicing regulator, TRA, to create a functional, female-specific isoform (Salz and Erickson 2010). Although we did not observe extensive cross-regulation at the gene expression level (Supplemental Fig. 6), our data do indicate cross-regulation of splicing among the 56 RBPs studied. Specifically, depletion of 31 of the RBPs affected the splicing of at least one of the 56 RBP pre-mRNAs, and splicing of 26 of the RBP-encoding pre-mRNAs was affected by depletion of at least one of the 56 RBPs (Fig. 2). The greatest extent of cross-regulation was observed upon depletion of PSI and CAPER, which each affected the splicing of six of the 56 RBP pre-mRNAs. On the other extreme, splicing of the *Syp* and *Rm62* pre-mRNAs were each affected by the depletion of 11 different RBPs. The cross-regulation of CAPER on splicing of other RBP genes and the splicing regulation of *Syp* by other RBPs was confirmed by RT-PCR (Supplemental Fig. 2A).

### Most SR and hnRNPs act coordinately rather than antagonistically

**Figure 3.**
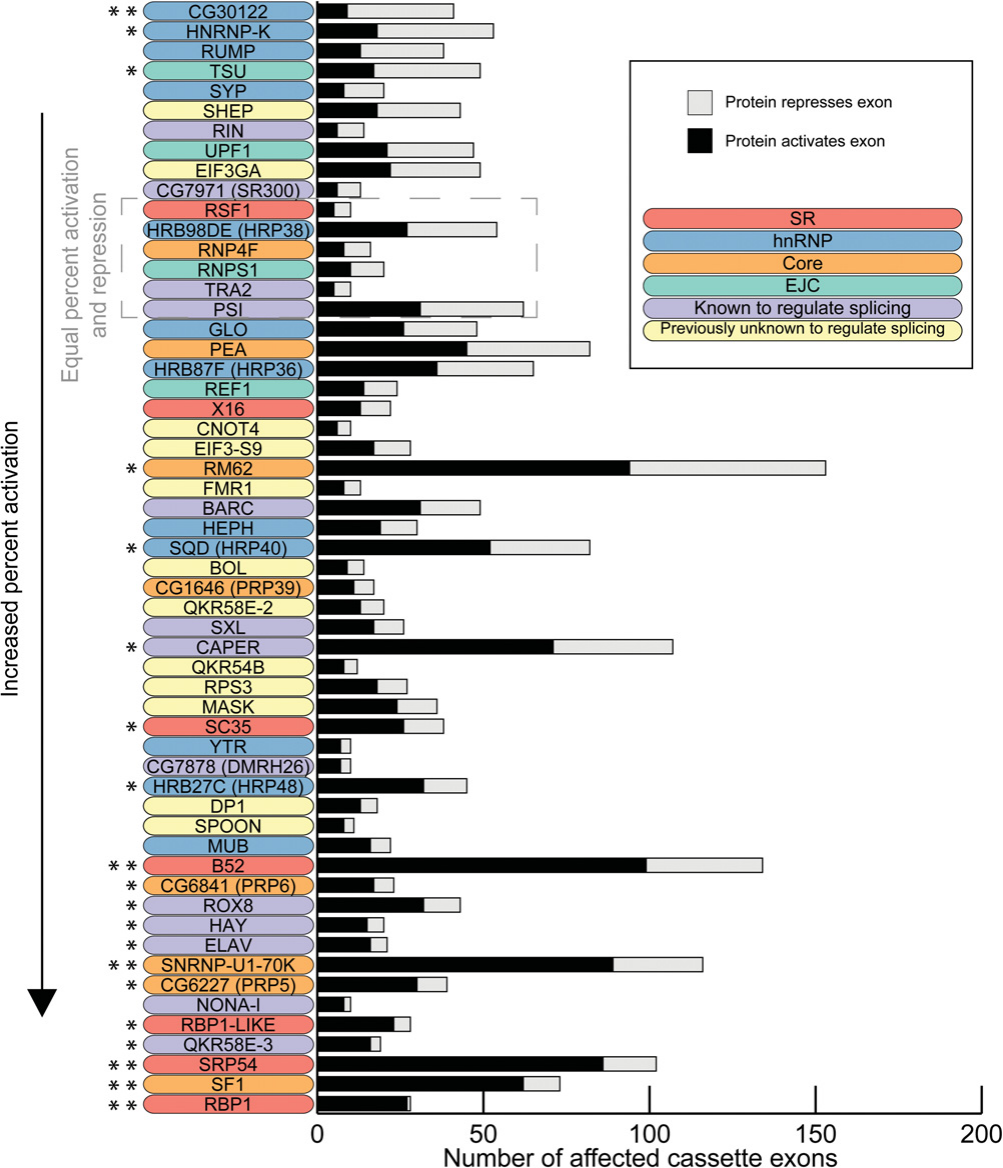
Bias of activation and repression of cassette exons. The number of cassette exons that are activated and repressed by each protein is shown. The proteins are ordered by increasing proportion of activated exons. The significance of the bias to activate or repress exons is indicated with asterisks: two-sided binomial test; (*) *P* < 0.05, (**) Bonferonni-corrected *P* < 0.05.

Many previous studies have shown that SR proteins tend to activate splicing while hnRNP proteins tend to repress splicing, and therefore, we examined the activity biases among the 56 proteins (Fig. 3). We infer that when an exon is excluded upon knockdown of a protein, that protein functions to activate splicing of the exon, and conversely, repression is inferred when increased inclusion of an exon is observed upon knockdown of a protein. Although SR proteins preferentially promote exon inclusion—B52, RBP1, and SRP54 strongly promoted exon inclusion (binomial test, Bonferroni-corrected *P* < 0.05)—hnRNP proteins had more varied effects. For example, SQD (HRP40) and HRB27C (HRP48) had a significant preference to promote exon inclusion, while CG30122 and HNRNP-K promote skipping (binomial test, *P* < 0.05). SF1 and SNRNP-U1-70K, core components of the spliceosome, strongly promoted cassette exon inclusion (binomial test, Bonferroni-corrected *P* < 0.05). In general, most proteins had a tendency to activate splicing, 10 proteins (mostly hnRNP proteins) repressed exons, and six showed no bias.

**Figure 4.**
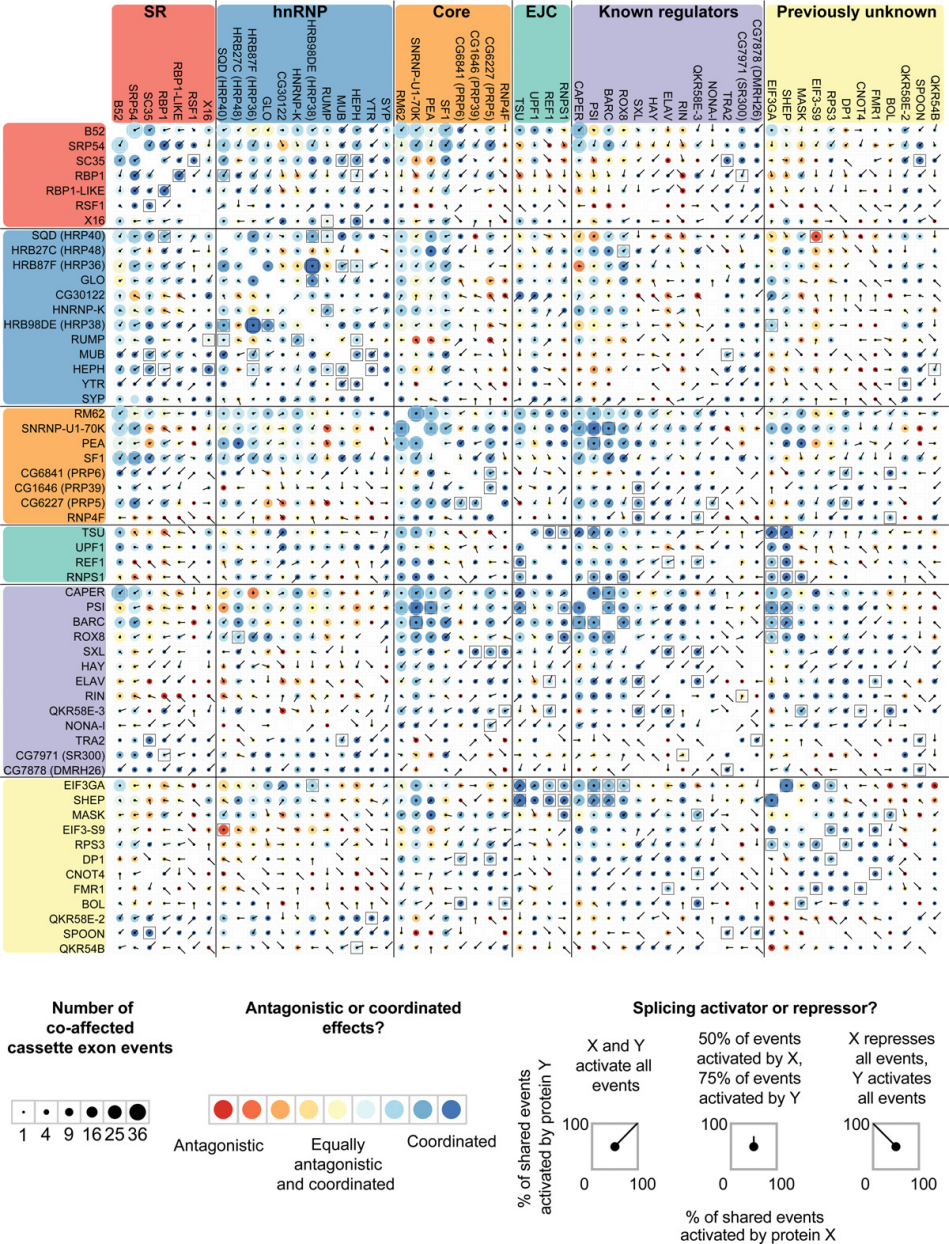
Antagonistic and coordinated effects of splicing regulators. Comparisons of cassette exons coaffected by each pair of proteins are represented as a matrix. Within each group (e.g., SR, hnRNP, Core, etc.), the proteins are ordered by the number of splicing events they affect, as shown in Figure 1. Information in the *upper* and *lower* portions of the matrix is identical. The size of each circle gives the relative number of coaffected events, and the color indicates if the pair acted antagonistically (one protein activates, the other represses) or coordinately (both proteins activate or both proteins repress). Overlaid spokes indicate what proportion of shared events is activated or repressed by each protein in the pair, thus giving more detailed information on the nature of the antagonistic or coordinated effects. Pairs of proteins that have a significant overlap in the number of coaffected events are indicated with black squares (Fisher's exact test, Bonferroni-corrected *P* < 0.05).

We next examined the extent to which individual splicing events are regulated by multiple proteins and, when multiple proteins affect a single splicing event, whether they act antagonistically or coordinately. The majority of splicing events (56%) are significantly affected by more than one protein (Supplemental Fig. 7). One pair of proteins that had a large number of overlapping effects is SNRNP-U1-70K and PSI, which physically interact (Labourier et al. 2001) and work coordinately to regulate P-element splicing (Siebel et al. 1992). We observed that in nearly all cases, SNRNP-U1-70K and PSI work coordinately to regulate splicing of the target mRNA in the same direction. Among all pairs of proteins, we identified 81 pairs that had a statistically significant overlap in the cassette exon events that they affected (Fisher's exact test, Bonferroni-corrected *P* < 0.05) (Fig. 4, black squares). One example of a significant overlap in the exons affected by two proteins is HRB98DE (HRP38) and GLO, which also physically interact (Guruharsha et al. 2011). Another example involves the previously uncharacterized splicing regulators, EIF3GA and SHEP, which had a significant overlap in their regulatory targets. Additionally, both RNA binding proteins had overlapping targets with multiple proteins in the EJC complex (particularly TSU), which have previously been shown to regulate splicing (Ashton-Beaucage et al. 2010; Roignant and Treisman 2010; Michelle et al. 2012). These genetic interactions suggest possible physical interactions among EIF3GA, SHEP, and the EJC complex; however, biochemical studies are necessary to confirm this. Coordinate regulation of splicing is known to be context specific (Ke and Chasin 2011); therefore, we may be missing additional coregulated splicing events that occur in a different cell type.

Our observation of cross-regulation complicates our analysis of coregulated targets. It is difficult to assess whether observed splicing changes are secondary effects of cross-regulation where knockdown of an RNA binding protein affects splicing or expression of another RNA binding protein. To address this, we identified cases where RNAi depletion of one protein's gene (gene A) significantly down-regulated the gene expression or affected splicing of another RBP gene (gene B) (Fig. 2; Supplemental Fig. 7). We then asked if there was a significant overlap in the observed splicing changes upon knockdown of gene A and gene B (Fig. 4, black squares). There were no cases where a significant overlap of splicing changes between knockdown of gene A and gene B could be explained by the down-regulation of gene expression or change in splicing of B. Therefore we do not believe that coregulated splicing events are due to secondary effects from changes in the other RNA binding proteins assayed; however, we cannot rule out secondary effects from other splicing regulators not targeted in this study.

In contrast to previous reports (Mayeda and Krainer 1992; Caceres et al. 1994), we found that in situations where a cassette exon was affected by both an hnRNP and an SR protein, the proteins tended to act coordinately to promote exon inclusion (Fig. 4). SR proteins tended to act coordinately with each other to activate exons (Fig, 4, top left corner), while hnRNP proteins also tended to act coordinately but, in many cases, to either activate or repress exons. For example, HRB98DE (HRP38) and GLO shared 16 events and they activated and repressed a similar number of exons, but they did so in the same direction in 14 of those cases (Fig. 4). There were only a few antagonistic SR and hnRNP protein pairs where the SR protein tended to activate and the hnRNP protein tended to repress, such as the strong exon activators SRP54, RBP1, and RBP1-LIKE with the strong exon repressors CG30122 and HNRNP-K (Figs. 3, 4). Therefore, our results do not support a general model of antagonistic regulation between SR and hnRNP proteins.

## Discussion

Together, this work provides a valuable resource of splicing regulatory networks in *Drosophila*. We identified approximately 3000 individual splicing events affected by knockdown of one or more of these 56 proteins. For 12 of the RNA binding protein genes (*Srp54*, *CG6227*, *Rm62*, *mub*, *qkr54B*, *Upf1*, *B52*, *Rbp1*, *elav*, *snRNP-U1-70K*, *Syp*, and *SC35*), a separate study found a significant overlap between the regulated splicing events reported here and binding targets of the same protein using RIP-seq (Stoiber et al. 2015). This suggests that the splicing events regulated by these 12 proteins are more often due to primary effects. Stoiber et al. (2015) also examined two other RNA binding proteins, TRA2 and FRM1, but they did not have a significant overlap of RIP-seq targets and regulated splicing events, suggesting indirect effects or additional nonsplicing related functions. For the remaining 42 proteins, additional studies are necessary to test whether each protein directly binds to the pre-mRNAs that are regulated to help establish primary versus secondary effects—particularly in light of the cross-regulation observed among RBPs and effects on AFE events.

This work, as well as others (e.g., Huelga et al. 2012; Bradley et al. 2015), further demonstrates that SR and hnRNPs both promote exon inclusion and exclusion; although in some cases we see a significant bias toward one mode of regulation. Moreover, our study does not support a general model of antagonistic regulation between the SR and hnRNP proteins. We also identified specific splicing events affected by depletion of core components of the spliceosome and components of the EJC, further supporting their function in alternative pre-mRNA processing.

We would not expect the target genes of the *D. melanogaster* splicing regulators to be broadly conserved in distant eukaryotes; however, there is evidence that regulatory specificity of *Drosophila* splicing regulatory proteins extends to orthologs in other eukaryotes, such as human and mouse (Brooks et al. 2011; Irimia et al. 2011). We found multiple genes in our study, such as SHEP and EIF3GA, that have not been previously characterized as regulators of splicing. It is possible that the splicing regulatory functions of these genes are conserved across metazoans.

## Methods

### RNAi depletion and RNA-seq

S2-DRSC cells were treated twice with 20 µg of dsRNAs individually targeting each RNA binding protein gene in biological replicates. After 5 d, total RNA was harvested and used to prepare poly(A)+ mRNA-seq libraries, which were sequenced on an Illumina GAIIx to generate single end reads of 75–76 bp. Reads were simultaneously aligned to the genome and splice junctions using Bowtie (Langmead et al. 2009). Additional details are described in Supplemental Methods.

### Splicing analysis

#### Identifying alternative splicing events

Although JuncBASE (Brooks et al. 2011) is designed to identify differential splicing events, it can also be used to identify all possible alternative splicing events from genes that were observed to be expressed. As coverage across a gene can vary in RNA-seq data (Hansen et al. 2010; Li et al. 2010; Kakaradov et al. 2012), an expression cutoff was made on individual splicing events (20 reads; total of reads supporting the inclusion isoform and exclusion isoforms).

Although a splice junction can be observed as constitutive in the observed RNA-seq data, it may have the potential to be alternatively spliced in other contexts; therefore, alternative splicing events were identified by including potentially unexpressed splice junctions that were annotated in MB8 or MDv1 (Graveley et al. 2011) that provide evidence that expressed constitutive exon–exon junctions can be alternatively spliced. For example, if an expressed junction differs in the corresponding 3′ splice site of an annotated junction, this gives evidence that the expressed junction has the potential for alternative splicing. The coordinates of expressed splice junctions and additional annotated junctions giving evidence of alternative splicing were formatted into BED files, which are created in a preprocessing step of JuncBASE. The BED files were then directly used as input to JuncBASE to identify and classify all possible splicing events.

Briefly, JuncBASE uses splice junction alignments and exon coordinates to identify the following alternative splicing events: cassette/skipped exons (SEs), A5SSs, A3SSs, mutually exclusive exons (MXEs), coordinate cassette/skipped exons (CSEs), AFEs, alternative last exons (ALEs), and retained introns (RIs) (Supplemental Fig. 8).

Exon coordinate annotations from the MDv1 annotation (Graveley et al. 2011) and novel exons identified through Cufflinks v0.9.3 (Trapnell et al. 2010) were used as input to JuncBASE. Each alignment file from the RNAi samples and the untreated sample was run through Cufflinks to identify de novo transcript annotations using the option -I 200000.

JuncBASE restricts the classification of A5SSs and A3SSs to events near annotated exons; however, this restriction was not enforced in cases where an alternative junction was identified in MDv1.

AFEs were identified by rerunning JuncBASE using only first exon annotations in FlyBase r5.32 with CAGE tag evidence. AFE events called from this run were combined with all other AS types from the JuncBASE run using the MDv1 annotation and de novo transcripts, described above.

#### Alternative splicing quantification

After the identification of each alternative splicing event, JuncBASE counts reads supporting the inclusion and exclusion isoform of each event. Isoform abundances are then calculated by dividing the read counts for the isoform by the length of the isoform. Ψ-values for each splicing event are derived from the isoform abundances:

$$\Psi =\;\frac{\mathrm{inclusion}\;\mathrm{isoform}\;\mathrm{abundance}}{\mathrm{inclusion}\;\mathrm{isoform}\;\mathrm{abundance}+\mathrm{exclusion}\;\mathrm{isoform}\;\mathrm{abundance}}.$$

#### Virtual reference calculation

To control for any splicing changes that may have been caused as an artifact of performing RNAi, a virtual reference sample was created to serve as a control. The assumption behind the creation of a virtual reference is that most splicing events will only be affected by a subset of proteins; therefore, comparing splicing in an RNAi sample with the median splicing across all events will identify splicing events specifically affected by each protein. The virtual reference exclusion and inclusion counts were calculated from the median expression and median Ψ values from all RNAi samples:

virtualreferenceinclusioncount=mediantotalcountofevent×medianinclusionratio;

virtualreferenceexclusioncount=mediantotalcountofevent×medianexclusionratio.

#### Differential splicing calculation

A Fisher's exact test was performed for each splicing event comparing the exclusion and inclusion read counts from each RNAi sample to the virtual reference inclusion and exclusion counts to identify samples with a significant difference in splicing. For each RNAi sample, a Benjamini-Hochberg multiple testing correction for each event type (e.g., cassette exon, A5SSs, etc.) was performed to give a final set of affected splicing events with a FDR < 0.05.

Alternative splicing events with more than two isoforms were tested for significant differences in splicing by treating each of the isoforms as the inclusion isoform and all other combined isoforms as the exclusion isoforms. In the final reporting of significantly affected events, the inclusion isoform giving the lowest corrected *P*-value is reported.

### Gene expression

Cufflinks and CuffDiff (Trapnell et al. 2010) were used to quantitate gene expression levels using the MDv1 annotation (Graveley et al. 2011). To quantitate expression levels of the 56 targeted RNA binding proteins, the annotation was masked of dsRNA regions used for the RNAi depletion to disambiguate reads that may have originated from either dsRNA or the target gene.

### Identifying target genes of transcription factors

To identify target genes of transcription factors, we used the peak-finding results from ChIP-seq data sets for 41 transcription factors (Negre et al. 2011; Boyle et al. 2014). We identified transcription factors that had ChIP-seq peaks 2000 bp upstream up to 100 bp downstream from the transcription start (according to the MDv1 annotation), and produced a TFBS score for each transcription factor and transcript and gene based on peak coverage. From the distribution of TFBS scores, we selected a cutoff of high TFBS scores to define transcription factor target sites.

## Data access

The sequence data from this study have been submitted to the NCBI Gene Expression Omnibus (GEO; http://www.ncbi.nlm.nih.gov/geo/) under sample accession numbers GSM461183–GSM461210 and GSM627333–GSM627418.

## Supplementary Material

Supplemental Material
