# Supplementary material for: Regulation of alternative splicing in Drosophila by 56 RNA binding proteins
Source: Genome Res. 2015 Nov;25(11):1771–80. doi: 10.1101/gr.192518.115 (PMC4617972; doi:10.1101/gr.192518.115)
Supplement: Supplemental Material [file supp_25_11_1771__index.html]

Regulation of alternative splicing in Drosophila by 56 RNA binding proteins — Regulation of alternative splicing in Drosophila by 56 RNA binding proteins — Supplemental Material 

# Regulation of alternative splicing in *Drosophila* by 56 RNA binding proteins

## Supplemental Material

**Files in this Data Supplement:**

- Supp Material.doc
- Supp Table 1.xlsx
- Supp Table 2.xlsx
- Supp Table 3.txt
- Supp Table 4.txt
- Supp Table 5.txt
