# Supplementary material for: Regulation of alternative splicing in Drosophila by 56 RNA binding proteins
Source: Genome Res. 2015 Nov;25(11):1771–80. doi: 10.1101/gr.192518.115 (PMC4617972; doi:10.1101/gr.192518.115)
Supplement: Supplemental Material [file supp_gr.192518.115_SuppMaterial.doc]

Supplemental Information

for

Regulation of alternative splicing in *Drosophila* by 56 RNA binding proteins

Angela N. Brooks, Michael O. Duff, Gemma May, Li Yang, Mohan Bolisetty,
Jane Landolin, Ken Wan, Jeremy Sandler, Susan E. Celniker,
Brenton R. Graveley^*^, Steven E. Brenner^*^

*Corresponding Authors

##

### Supplemental Figure 1: RT-PCR validations of RNAi knockdowns. a. RT-PCR was performed on all samples using primers to RP49 as a control for RNA quantities. b. The efficiency of depletion was monitored RT-PCR of the target gene in both replicates of the RNAi samples and compared to the levels in both replicates of the untreated control samples.

###

### Supplemental Figure 2: Correlation between JuncBASE ΔPSI, a. RT-PCR ΔPSI, and b. Bradley *et al.* ΔPSI. Scatter plot of the ΔPSI as calculated by JuncBASE from the RNA-seq data, a. from the RT-PCR reactions or b. reported in Bradley et. al. 2015. Values for splicing events significantly affected by the knockdown of one or more proteins as determined by JuncBASE are shown. A best fit regression line is shown with R^2^ based on Spearman.

### Supplemental Figure 3: Depletion efficiency/specificity of each RNA Binding Protein Sample. The FPKM of all 56 RNA Binding Protein genes was calculated in each sample and the depletion efficiency calculated as [(FPKM_untreated_ - FPKM_knockdown_)/ FPKM_untreated_]. The results are plotted as a heatmap with the RNAi samples in columns and the RNA Binding Protein genes in rows. The bright diagonal indicates efficient depletion of the target gene.

### Supplemental Figure 4: Significant changes in gene expression observed upon knockdown of 56 proteins. The fold change in FPKM levels of all was calculated in each RNAi sample in comparison to the untreated control sample. The number of genes that were significantly affected and the magnitude of those changes are plotted for each sample.


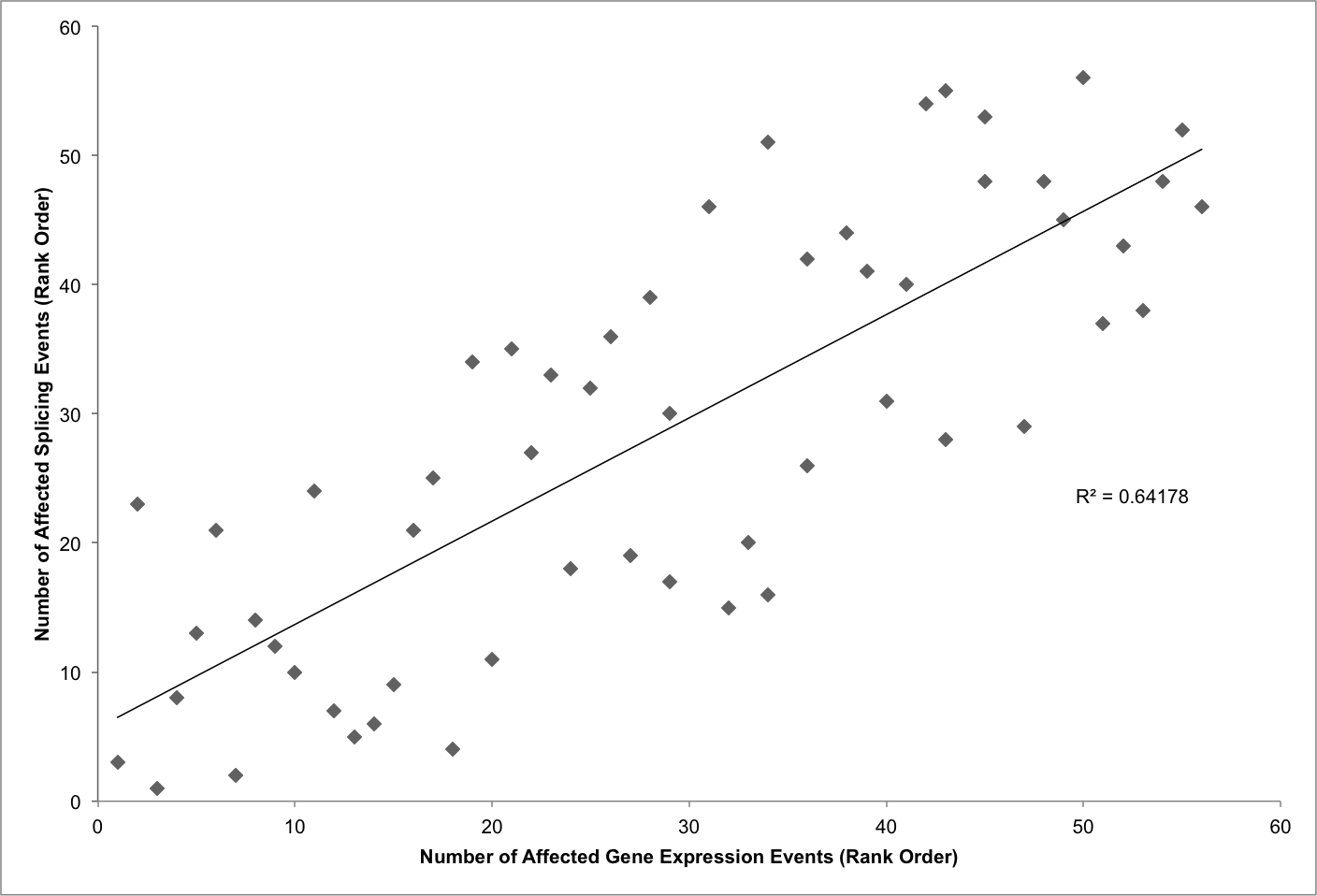


### Supplemental Figure 5: Comparison of the number of gene expression and splicing events affected in each sample. For each protein, the number of gene expression and splicing events that were significantly affected was calculated and plotted as the rank order.

### Supplemental Figure 6: Cross regulation of gene expression between the 56 RNA binding proteins. The fold change in FPKM levels of each of the 56 RNA binding proteins tested was calculated in each RNAi sample in comparison to the untreated control sample and plotted as a heatmap representation. Fold change of target gene is not shown and is colored grey.

###

### Supplemental Figure 7: Specific and shared effects by 56 proteins. For each class of splicing event, the number of events that were affected be each number proteins is plotted. Most events are only affected by one protein, but many are affected by more than one.

**Supplemental Figure 8: Schematic depiction of the various classes of alternative splicing events.** The various classes of alternative splicing events are depicted.

## Supplemental Tables

### Supplemental Table 1: RNA Binding Proteins Studied. The RNA binding proteins analyzed in this study are listed. The gene names (CG number and synonyms), domain types, category, and number of uniquely aligned reads are indicated. Additionally, the ranges of correlation of exon coverage from RNA sequencing lanes between biological replicates are shown. The categories are not mutually exclusive. Core; core component of the spliceosome. EJC; exon junction complex or nonsense-mediated decay. Prior, No Prior; Prior or no prior evidence for a role in splicing regulation.

### Supplemental Table 2: Primers used for RT-PCR Validation of RNAi Depletion

### Supplemental Table 3: Annotation and PSI values for all splicing events observed from RNA-seq

### Supplemental Table 4: Annotation of all splicing events considered significantly affected by at least one of the 56 proteins. Percent spliced in (Psi) values are given only for samples with differential splicing from reference, otherwise “NA” is given.

**Supplemental Table 5: Significant changes in gene expression upon knockdown of 56 proteins.**

## Supplemental Methods

### Identifying *D. melanogaster* proteins with an RRM or KH domain

All *D. melanogaster* protein sequences were obtained from Uniprot . Each sequence was searched against the Pfam database using hmmpfam (hmmer.org) for the presence of the Pfam domains RRM_1, RRM_2, RRM_3, KH_1, KH_2 with the default cutoff score. To find additional proteins that may have been missed by Pfam, each sequence was also compared against SMART domains RRM, RRM_1, and KH and against the Prosite domains RRM (PS50102), KH_TYPE_1(PS50084), KH_TYPE_2(PS50823). The SMART hmms were scanned with hmmsearch (hmmer.org) and Prosite domains with the local version of ScanProsite, ps_scan.pl .

### Determining expression of putative splicing regulator genes from Affymetrix tiling array data

Before selecting target genes, we checked for their expression in S2-DRSC cells. S2-DRSC mRNA expression data was available from 38 bp Affymetrix tiling arrays . Transcribed fragments (transfrags) from the data were selected using a bandwidth of 0, maxgap 90, and minrun 50. Genes with at least 10% transfrag coverage were considered expressed. For genes not passing the transfrag coverage cutoff, probe intensities were reviewed manually and additional genes were called expressed.

### RNAi depletion

RNA interference was performed essentially as described previously . Vectors encoding double-stranded RNAs for the target mRNAs were generated as described previously . Briefly, cDNA fragments encoding the specific dsRNA were amplified by RT-PCR with gene-specific primers from total RNA isolated from S2-DRSC cells, cloned into the pCRII-TOPO vector (Invitrogen), and sequenced to verify the identities of the inserts. DNA templates were amplified with M13 forward and M13 reverse primers and the PCR products were used in individual *in vitro* transcription reactions with the Ampliscribe High Yield Transcription SP6 (Epicentre) kit and T7 kits (Epicentre) to generate the sense and antisense RNA strands. After DNase I digestion, the single-stranded RNAs were annealed to generate dsRNAs. Integrity of the PCR products, the single-stranded RNA transcripts, and dsRNAs were monitored by agarose gel electrophoresis.

S2-DRSC cells (obtained from the Drosophila Genomics Resource Center at Indiana University) were cultured with Schneider’s medium (Sigma/Aldrich) plus 10% heat-inactivated fetal calf serum (FCS) (HyClone) at 27°C. One day prior to dsRNA treatment, cells were split into six-well culture dishes at a density of 1X10^6^ cells/mL. Immediately prior to the addition of dsRNA, the culture medium was replaced with fresh Schneider’s medium without FCS, followed by the addition of 20 µg of each dsRNA directly into the FCS-free medium and the cells incubated for 5 h at 27°C. After incubation with the dsRNA, 10% FCS was added back to cell culture. After 2 d, a second dose of 20 µg of dsRNA was added to each well in the same manner as described above and the cells incubated for two additional days after the re-addition of 10% FCS. After the dsRNA treatment, total RNA was isolated using TRIzol reagent (Invitrogen) according to the manufacturer’s directions. Parallel dsRNA treatments and total RNA preparations were performed independently for each replicate. Untreated S2-DRSC cells were used as a reference. To monitor the level of mRNA depletion, primer sets (Supplementary Table 2) that amplify regions of the targeted mRNAs outside of the dsRNA region were used for RT-PCR amplification, and compared with the results from the untreated cells (Supplemental Figure 1).

**Sequences of dsRNAs used for RNA Interference**

>glo:

GTGAAGCTTCGTGGTCTGCCATATGCCGTCACTGAGCAGCAAATCGAGGAGTTCTTCTCTGGGTTGGATATCAAAACGGATCGGGAGGGCATACTTTTTGTTATGGACAGAAGGGGTCGTGCAACTGGGGAAGCTTTTGTTCAGTTCGAAAGCCAGGACGACACTGAGCAAGCCTTGGGCCGAAATCGGGAAAAAATTGGGCACAGGTATATTGAGATATTCCGCAGCTCGATTGCTGAAATGAAGAGGGCCACAGGCGCCGGTGGCGGTGTCGGAGGACGCCCTGGCCCTTATGACATACGTGATCGTGGTGC

>rump:

ATACGACTACCGTTGGCAGGATCTGAAGGATCTGTTCCGCCGCATCGTCGGCTCCATTGAGTACGTCCAGCTGTTCTTCGATGAGAGCGGCAAGGCTCGCGGCTGTGGCATCGTAGAGTTCAAGGATCCGGAGAACGTACAGAAGGCCTTGGAGAAAATGAACCGCTATGAGGTGAATGGCCGCGAACTGGTGGTCAAGGAGGATCACGGCGAGCAGCGCGATCAATACGGACGCATTGTGCGAGATGGTGGTGGTGGTGGAGGCGGCGGTGGCGGCGTACAAGGAGGCAATGGTGGCAACAATGGAGGAGGTGGCGGCGGTGGCCGTGACCACATGGATGACCGCGATCGGGGTTTCTCCCGGCGAGACGACGACAGACTATCTGGGCGTAATAATTTTAACATGATGTCAAATGATTATAATAATTCGTCGAATTACAATTTGTATGGGCTTTCTGCTTCGTTTTTG

>msi

GAAGGTCGAGTGCAAGAAGGCACAGCCCAAGGAAGCAGTCACACCGGCTGCTCAGCTTCTCCAGAAGCGCATTATGTTGGGCACCCTCGGCGTCCAGCTGCCCACAGCTCCTGGCCAGCTGATTGGAGCCCGTGGTGCCGGCGTGGCCACCATGAACCCACTGGCCATGCTTCAAAATCCCACACAGCTACTGCAATCCCCGGCAGCAGCCGCTGCCGCCCAGCAGGCCGCCCTCATATCACAGAACCCATTTCAAGTACAAAACGCCGCTGCGGCAGCCTCGATTGCCAATCAGGCTGGCTTCGGCAAGCTGTTGACCACATATCCGCAGACTGCGCTGCATAGCGTCAGATATGCACCCTACTCGATCCCCGCCAGCGCCGCCACTGCCAACGCCGCCTTGATGCAGGCTCATCAGGCGCAAAGCGTGGCCGCCGCTGCCCATCATCACCAGCAGCAGCAACAGCAGCAGCATCATCAC

>Hrb98DE

AACTACGGCAACCAGAATGGTGGCGGCAACTGGAACAACGGTGGCAACAACTGGGGCAACAACCGCGGGGGTAACGACAACTGGGGCAACAACAGCTTCGGTGGTGGCGGCGGCGGCGGTGGTGGTTATGGCGGTGGCAACAACAGCTGGGGCAATAACAATCCGTGGGACAATGGCAATGGAGGCGGCAACTTTGGAGGCGGCGGCAACAATTGGAACAATGGTGGCAATGATTTTGGAGGCTACCAGCAGAACTATGGCGGCGGTCCGCAGCGAGGTGGCGGCAACTTCAACAACAATCGCATGCAGCCCTACCAAGGAGGTGGTGGATTCAAAGCAGGCGGTGGCAATCAAGGCAACTATGGCGGAAACAATCAGGGCTTCAATAACGGTGGC

>Hrb87F

CTACCGCACCACAGATGATGGCCTGAAGGCTCACTTCGAGAAGTGGGGCAACATTGTCGACGTGGTGGTGATGAAGGATCCCAAGACGAAGCGCTCTCGCGGCTTCGGTTTCATCACGTACTCCCAGTCGTACATGATCGACAATGCGCAGAATGCCAGGCCACACAAGATCGATGGACGCACCGTGGAGCCCAAGAGGGCTGTGCCACGCCAGGAGATCGATTCCCCGAATGCGGGAGCCACGGTAAAGAAGCTCTTTGTGGGCGGGCTTCGAGACGATCACGATGAAGAGTGCCTGCGCGAGTACTTCAAGGACTTTGGCCAGATCGTGAGCGTGAACATTGTTTCCGACAAGGACACCGGCAAGAAGCGCGGCTTCGCCTTCATTGAGTTC

>Syp

GACAGTTCCTGGAATCGAACCTGGAGCACGTGTCAAACAAGTCCGCCTACCTATGCGGCGTGATGAAGACGTACCGACAGAAGAGTCGAGCCAGCCAACAGGGCGTGGCCGCGCCCGCAACTGTCAAAGGTCCCGACGAGGACAAGATCAAGAAAATCCTCGAGCGCACCGGCTACACATTAGATGTGACGACAGGTCAGCGTAAATACGGCGGACCGCCGCCGCATTGGGAGGGAAATGTGCCAGGCAACGGTTGCGAGGTTTTCTGCGGCAAGATACCCAAGGACATGTACGAGGACGAACTGATTCCGCTATTCGAGAACTGCGGCATAATCTGGGACCTACGACTCATGATGGACCCGATGACGGGCACAAATCGTGGTTATGCATTTGTCACATTCACAAATCGCGAAGCGGCCGTCAATGCAGTGCGACAGCTCGATAATC

>sqd

GGAAACTGTTTGTCGGTGGTCTGAGCTGGGAAACGACTGAGAAGGAACTCCGCGATCACTTCGGCAAATATGGCGAGATCGAGAGCATCAATGTCAAGACAGATCCCCAGACCGGTCGGTCCCGAGGATTCGCCTTCATCGTGTTTACAAACACCGAGGCCATTGACAAAGTCAGCGCCGCGGATGAGCACATAATCAACAGCAAGAAGGTCGATCCCAAGAAGGCCAAGGCCAGGCACGGCAAGATCTTTGTCGGCGGCCTCACCACAGAGATCAGCGATGAGGAGATTAAGACCTACTTTGGACAGTTCGGCAATATCGT

>HnRNP-K

AAATACTTTGAGGAGCGCGACGAGGACTTTGATGTGCGTCTACTTATACACCAGAGCTTGGCCGGCTGCGTCATTGGCAAAGGTGGACAAAAGATCAAGGAGATCCGCGATCGCATCGGCTGCCGCTTTTTGAAGGTCTTCTCGAATGTGGCACCACAGAGCACAGATCGAGTGGTGCAGACCGTTGGCAAGCAGAGCCAGGTCATCGAAGCGGTTCGTGAGGTGATCACACTTACACGGGACACTCCCATCAAGGGGGCGATACATAACTATGATCCTATGAACTTTGACCGCGTATATGCCGATGAGTACGGTGGCTATGGC

>CG30122

GATGAAGGTGGTGGACCTGCGCAACGAGCTCCAGTCGCGCGGCCTGGACACCAAAGGAGTCAAAGCGGTGCTCGTCGAGCGCCTGAGGGCATATGTGGAAGGAGGAGCCGGCGACGGTGAAAATGCGCCGGTCACACCAAGCCGCCGTCAGCGTCGCACGCGCTCTATGTCCCGCTCTCCATCGCCGGTGCAAGCTGCTCCCGTGGCCGCAGAACCAGTGCTCGATACTCTCGAAGAGGAGGAGCAGCCGGAGGATAAGACAGTGCCACAGCCAGAACCAGAAAGTGAACAGCCAGCAGCCGAGCCGGAACCAGAACAAAGTGAGCCGGAGGAAGCTGAGCCAGCTGCAGCAGTGACAGAGGACACAACCGTCAACCAAG

>Srp54

CTTGACCAACACGGTGTTCATCGATCGCGCCCTAATTGTCATACCCGTTCTGGCCATACCCGAGGAGTATCGGGCCCTGGAGATGCTCAAGAACGGAACCATTGTGCCGGGACTCCAGAAGCCGGACTCCAAGCTACCGCCCGAAGTCATTAACCGCATCGAGGGACAGCTGCCGCAGCAAGTGATCAAGACGTACGACCCCAAGTTGGTGGAATTCAATCTGCCGGAGTACCCGGCCTTACCCTCGTTCTACGATGCGCGCAAAATCGAGGAGATTCGGCGCACCATTATCGTGTGCGATGTTAAGAACGAGTGGCGGCTAGACGATCTGATGGAATGCTTTCAGCGCGCTGGGGAGGTGAAGTATGCCCGTTGGGCCGAGAAGGATAACAAGACGTACTGCATGATTGAGTTCTGCGAACAGACCAGCATTATTCACGCCCTGCGCATGCAGGGCCAGGAGTTCAAGGGTGGCCAT

>Rsf1

GTTCACAAAGTATGGCAAGCTGAATTCGGTGTGGATAGCCTTCAATCCGCCGGGATTTGCGTTCGTCGAGTTCGAGCACCGCGACGACGCCGAAAAGGCGTGCGACATACTGAACGGATCCGAGCTGCTCGGCTCCCAGCTGCGCGTGGAGATCTCAAAAGGGCGGCCACGCCAGGGTAGGCGTGGCGGACCCATGGACAGGGGCGGACGACGCGGCGACTTTGGCCGGCACAGCATCACAAGCGGTGGTAGCGGCGGAGGCGGTTTCCGGCAGCGCGGATCCAGCGGATCCTCAAGCCGGCACACGGAGCGGGGCTATAGCTCCGGCCGATCAGGTGCAAGCAGCTATAATGGCAGAGAGGGCGGCGGCAGCGGCTTCAATCGCCGCGAGGTTTACGGCGGTGGACGCGACAGCAGCCGCTACAGCAGCGGAAGTAG

>Rbp1-like

ACAAGTCCAGTGGTACAACAAATACCAAAAATCCATTACAGAACCGGAGGAGCAGCACCTCCAGCCACATACATATTCATACATAATGCCACGCTACCGTGAATGGGATTTAGCCTGCAAAGTTTACGTGGGCAATCTGGGATCCTCGGCTCCAAATACGAGATCGAGAACGCCTTTAGCAAATACGGACCCTTGCGCAACGTCTGGGTGGCCCGCAATCCGCCCGGTTTCGCCTTCGTCGAGTTCGAGGATCGTCGCGACGCTGAGGATGCGACCCGTGGCCTCGACGGCACCCGCTGCTGTGGCACCCGCATCCGTGTCGAAATGTCATCAGGCCGTTCACGA

>x16

AAGGTGTACGTGGGCGATCTGGGCAACAATGCCCGGAAGAACGACCTGGAGTATGTATTTGGAGCGTACGGCAGTTTGCGCAGCGTCTGGATAGCCCGCAATCCGCCGGGCTTCGCCTTCGTGGAGTTTGAGAGTGCCCGCGATGCGGCGGATGCGGTGCGCGGATTGGACGGACGGACGGTTTGCGGGCGCCGAGCCCGTGTGGAATTGTCCACCGGAAAGTATGCTAGGTCCGGCGGTGGTGGTGGCGGAGGTGGTGGAGGCGGTGGTGGTGGAGGACTCGGAGGACGCGACCGAGGCGGCGGTGGTCGTGGGGACGATAAGTGCTACGAGTGCGGCGGACGGGGGCATTTCGCTCGCCACTGTCGCGAAAGGAAGGCCAGGCAGCGACGCAGAAGCAACTCATTCAGCAGATCTCGGAGCACATCGCGACGCAGGCGCACTCGCTCCAAGTCCGGAACTCGAT

>Rbp1

TGCCGCGATATAGGGAGTGGGACTTGGCCTGCAAGGTGTACGTGGGAAACCTGGGCTCCTCGGCGTCCAAGCACGAGATAGAAGGCGCATTTGCCAAATATGGACCCCTGCGAAACGTGTGGGTGGCCCGCAATCCACCAGGTTTCGCCTTTGTCGAATTTGAGGATCGCCGTGACGCGGAAGACGCAACGCGTGCCCTGGACGGAACACGCTGCTGCGGCACTAGGATTCGCGTAGAGATGTCTTCGGGTCGCTCGCGCGATCGCCGGCGCGGAGAAGGCGGCAGTAGTGGTCGCTCTGGTTCCGGACGCTACAGGTCACGTTCGCCACGTCGCTCCCGATCGCCCCGCAGCCGCAGCTTCTCGCGCGATCGTCGAAGTCGCTCGGATTCTCGGGATCGTCATTAA

>SC35

GGATCGCTACACACGTGAGAGCCGCGGATTCGCATTTGTTCGCTTCTATGACAAACGTGATGCCGAGGACGCACTGGAGGCCATGGATGGTCGCATGCTAGACGGCAGGGAGCTCCGCGTACAGATGGCCCGCTACGGACGCCCCTCTTCGCCCACTCGCAGCTCCAGTGGTCGTCGTGGCGGAGGAGGAGGCGGTGGTTCCGGCGGGCGTCGTCGGTCACGTTCTCGCTCCCCAATGCGCCGTCGTTCGCGCAGTCCGCGTCGCCGATCATACTCCCGTTCCCGCTCGCCTGGTAGCCACTCGCCGGAACGCCGATCCAAATTTTCACGCAGTCCAGTACGCGGCGACAGCCGCAATGGAATCGGAAGCGGATCTGGAGGACTGGCCCCAGCCGCGTCTCGTAGTCGCAGTCGCTCCTAGATATCGACGTCACGTTCCATTTAGTGGGAGTGCGAGATATGACTCGCTG

>B52

CATCAAAAATGGCTACGGCTTTGTGGAATTCGAAGACTATCGTGATGCCGACGATGCCGTCTATGAACTGAATGGCAAAGAGCTGCTTGGCGAACGTGTGGTTGTTGAACCCGCCAGGGGTACCGCTCGTGGCAGCAACCGCGACCGCTACGACGATCGATATGGTGGTCGGCGGGGGGGCGGGGGCGGTCGTTACAACGAAAAAAACAAAAATTCCAGATCATCCTCTCGTTATGGCCCACCGTTGCGCACTGAGTACCGACTGATTGTGGAGAATTTGTCTAGCCGCGTTAGCTGGCAGGATCTCAAGGATTACATGCGCCAGGCTGGCGAGGTCACCTATGCCGATGCCCACAAGCAGCGTCGCAATGAGGGCGTGGTTGAGTTCGCCTCGTTGTCGGACATGAAGACGGCCATTGAGAAGTTGGATGACACCGAGCT

>ytr

CCCAAATTCACAACAGAAAAATTTCCAAACTCACACACAGACAATTACTTAAAGTATTTGAAAACTTACCACAGCCCGAAACGCACTCTTCACATACCCATACCCTTATCCTTACCCATACCCATACCCATCCTCATCCGCATCCAGATCCCCATCCCGATCCCAACCGCAATCGAAAAGACCTCCCAACAAACTTCCTCCATACGCTGCAA

>CG7971

AAATCTCCAGCCCATTCCCCAGAGGCGCCACCGAAGAAGTCGGTGCCAACGCCAGCCTTCAATCCCTTTAAGGCGGCCGAGGATACTGTTAACGACATCCTTGGCACAAAGTCGGTGATGGTGGCCCTGGAACAGACTAAGCGACAGCGGGCGGCTTCCAGCTCTAGCTCGGATTCCGACAGCTCCGGTAGTAGCTCGACTTCCTCGCGTACGCCATCGCCTAAGCCCACACCTAGGAAACAAAAGAAGAGGAGCAAGACCCCAGAGCTAAAAGAGGTGAAGAAGGAGATTAGCCCCAGAAAGG

>tra2

AACAAGTACGGACCTATCGAACGCATCCAGATGGTGATTGACGCACAAACACAGCGTTCCCGGGGCTTTTGTTTCATTTACTTTGAGAAACTCAGCGATGCCCGCGCGGCTAAGGACAGCTGCTCCGGAATAGAAGTGGATGGTCGCCGTATTCGCGTCGATTTCTCTATAACCCAACGGGCTCATACCCCAACTCCGGGTGTGTATTTGGGTCGTCAGCCGCGTGGAAAAGCTCCACGCTCATTTTCACCGCGTAGAGGACGCCGTGTGTATCACGATCGCTCCGCTTCGCCCTATGACAACTATCGTGATCGCTATGATTACCGCAACGATCGCTACGACCGTAATCTCCGCAGGAGCCCTAGTCGCAACCGTTACACTCGCAACAGGAGCTACAGCCGTTCACGCTCTCCGCAACTACGTCGAACTTCATCGCGCTATTAAAGCGCCTGGGGAGGAGGCTACTTCATTAACTCGTGCTCCTAAGTTCGCCCAACT

>heph

GCGCGGAAGCGACGAACTTTTGAGTCAAGCAGCGGTCATGGCGCCCGCTTCCGACAATAACAATCAGGACCTGGCCACAAAGAAGGCCAAACTGGAGCCGGGCACTGTGCTGGCCGGCGGAATTGCCAAGGCCTCAAAAGTCATCCACTTGCGCAACATTCCGAACGAGTCCGGCGAGGCAGATGTGATTGCCCTGGGCATTCCGTTTGGACGTGTGACCAACGTGCTGGTGCTCAAGGGCAAGAACCAGGCTTTCATCGAGATGGCCGACGAGATCTCCGCAACGTCAATGGTGTCCTGTTACACAGTAACTCCGCCCCAGATGCGCGGCCGCATGGTCTACGTGCAGTTTTCTAATCATCGCGAACTAAAGACGGACCAAGGTCACAAC

>mub

ATCCATCGGTGACACTCACAATAAGGCTGATTATGCAAGGAAAGGAGGTTGGTAGTATTATTGGTAAAAAGGGTGAAATTGTCAACAGATTTCGTGAAGAGTCTGGTGCCAAAATCAACATTTCGGATGGCTCATGCCCGGAACGTATTGTGACTGTGTCTGGTACAACTAATGCAATCTTTTCGGCATTCACGCTCATTACAAAGAAGTTCGAAGAGTGGTGCTCGCAGTTCAATGATGTAGGCAAAGTTGGTAAAACTCAAATACCCATTCGATTGATTGTGCCCGCCAGTCAATGTGGATCGTTAATTGGCAA

>Upf1

GGAGGAGCTATGGAAGGAGAATATTGAGGCCACGTTTCAGGATCTGGAGAAGCCAGGCATTGACTCGGAGCCAGCACATGTGCTACTCCGCTACGAGGATGGCTATCAGTACGAGAAGACCTTTGGGCCGCTGGTCCGCCTTGAGGCCGAATACGACCAAAAACTGAAGGAGTCTGCCACGCAGGAGAACATCGAAGTACGCTGGGACGTCGGCCTCAACAAAAAGACCATTGCCTACTTTACGCTGGCGAAGACCGATTCGGACATGAAGCTCATGCATGGCGACGAGCTGCGCCTGCATTATGTGGGCGAGCTGTACAATCCGTGGAGCGAGATCGGCCACGTTATCAAGGTGCCGGACAATTTCGGCGATGACGTCGGCCTGGAGCTGAAATCCTCAACGAATGCCCCGGTTAAGTGCACCAGTAACTTTACGGTGGACTTCATCTGGAAGTGCACGTCATTTGATCGCATGACACGTGCTCTGTGCAAATTCGCCATCGA

>qkr58E-2

AGCATGAGAACGAACACAACGCCAACGCAGACGGCGAGAAGGCCCAGCCGGCGCCGGCGGTCCAGAAGTACATGCAGGAGCTCATGACGGAGCGATCGCGCATGGAAAACCACTTCCCCCTGGCGGTGAAGCTAATTGACGAAGCTCTGGAGCGTGTGCAGCTAAACGGACGCATTCCCACGAGAGACCAGTACGCCGATGTCTACCAGCAGCGCACCATCAAGCTGTCCCAAAAAGTGCACGTGCCCATCAAGGACAAGAAGTTCAACTATGTGGGCAAGCTACTGGGGCCCAAGGGCAACTCACTGCGTCGCCTGCAGGAGGAGACGCAGTGCAAGATCGTCATACTTGGTCGCTTCTCAATGAAGGATCGC

>SF1

CAGGATCACGATCAATCCAGAATCCCGTCGCTCTTCGACCGACAGCAGGGATTGGAAACCATCAGGGAGGAAGGACGTGAGCAGCGGTTTGATCTTACTCAGACCATCCAGGAGCTAATGGGCAATGCTGGAGGCAACAAAGGATTCGCCTCGTTCTTTAACAGCCAGAACAGCAACGACTCCACCAGCAATGGAGCATTCGATAACTCAGCGGACAGCGCTGCGGAGCGAAAGAGGAAGCGGAAGTCTCGCTGGGGCGGCAGTGAAAACGACAAGACCTTCATTCCTGGAATGCCCACAATTCTGCCCTCCACCCTGGACCCGGCACAGCAGGAGGCCTACCTAGTTCAATTTCAAATCGAGGAGATTAGTCGCAAGC

>spoon

CTGCCCGGTGTAGCATTTATACTCGGCGTCTTTTGGTTTCGGCGTAGATATAAAAATTGTTTAGACAAGCCCGACGACGAGGACTCATCGGCCATCAATGACTCGTCGATTGAACCAACTGTGCAGGCGCGCAAGGCCAACGGAGTCCTGCAGAATGGCAAGCTGCCACAGCAGTCGGCCAGCAAGTCGATGAACATCAACGGAACTTTAGTTAACGGTAGCGGAAGCGGTAGCGGAAGTAGCAGTGATGAGAAGGACAGCCCCACTACCATGTTGTATGGTAAATCAGCACCAATCAAAATCC

>CG7878

CACCAGGAGTACGTCGTTTAGCGCAGAGCTATATGAAGAATCCCATCCAGGTGTGTGTCGGATCGCTCGATCTGGCAGCCACGCACTCGGTGAAACAAATTATTAAATTGATGGAGGATGACATGGACAAATTCAACACCATTACATCTTTCGTTAAGAACATGTCCAGTACGGACAAGATCATCATATTTTGTGGACGCAAGGTTCGTGCTGACGACCTATCCAGTGAACTTACGCTGGATGGTTTCATGACCCAGTGCATTCATGGTAATCGCGATCAGATGGATCGTGAGCAGGCTATTGCCGATATTAAGTCCGGCGTCGTGCGCATTCTGGTTGCTACCGATGTGGCATCACGTGGCCTGGACATTGAGGATATCACACATGTCATCAACTATGATTTTCCGCACAACATCGAGGAGTATGTGCACCGTGTTGG

>mask

AACACAGGCTCTGGCTCTGGATCCAATAATAACAATAACAACACCAATCAAAACCCCAACAGACAGTTGAATCATAATTTACCCCGAATCGCTGCCGCCAGACAATCGATAGCCGCCGCTCTATTGAAAAACAGCGGGCGGAAGATTCTGACGGCCAAGAATGAGCCACTGACGACGACGGAGTCATCAGGCGTTTTAACCAACACACCTTTACCCAGCAATAGCCGATTGAAAGTTAACAACAACAACAACACCAATAACACTGCCAAGATGTCTGGAACTAGTAGCAGTCAGTCCTCGGCCACGCCCACACCGCCCACGGCCAGCAGCAGCACAACCACCACAACAACAACGAACATCAGCACCGGAGGCGGTGGGAGTGGCAGCAGTGGCGGTGGCGGTGGGAGTACCACGGTCATTGCCAATCCCGCATCGGTAACCAACACCGGAGCTGGAA

>Fmr1

AGACCGAGGAGTCTGTGCAGCGTGCCCGCGCGATGCTCGAATACGCCGAGGAGTTCTTCCAGGTGCCCAGGGAGTTGGTGGGCAAGGTGATTGGCAAGAATGGGCGCATTATCCAGGAGATTGTGGACAAGAGTGGCGTGTTTCGAATCAAGATCGCTGGCGACGATGAACAGGATCAGAACATACCACGTGAGCTGGCGCATGTACCCTTTGTGTTCATTGGCACCGTGGAGAGCATTGCAAATGCCAAAGTGCTGTTGGAGTATCATCTGTCGCACCTGAAGGAAGTAGAACAGTTGCGTCAGGAGAAGATGGAGATTGATCAGCAGCTTCGCGCCATCCAGGAATCCTCCATGGGCTCCACACAGAGCTTCCCAGTGACGCGGCGCTCTGAGCGCGGCTACAGCAGTGACATTGAGTCGGTGCGCTCTATGCGCGGCGGTGGTGGCGGCCAGCGTGGTCGTGTACGCGGACGTGGT

>Psi

CGTTATCATGTTGCGTGGTCAAAGGGATACAGTCACTAAGGGGCGCGAAATGATTCAGAACATGGCCAATCGGGCTGGCGGGGGACAGGTGGAGGTGCTGTTGACGATCAATATGCCGCCACCGGGACCTAGCGGGTATCCACCTTACCAGGAGATCATGATTCCGGGCGCCAAGGTGGGCTTGGTCATTGGCAAGGGCGGCGATACCATTAAACAGCTGCAGGAGAAGACCGGAGCCAAAATGATCATCATCCAGGACGGACCAAACCAGGAGCTGATCAAACCCCTTCGCATATCCGGCGAGGCGCAGAAGATAGAGCACGCCAAGCAGAT

>Dp1

CTACGAGGAGAACTTCACATTCGAGGTGATGACGGTTAATCCTTCGTACTACAAGCACATCATCGGTAAGGCTGGAGCCAACGTAAATCGCCTGAAGGATGAACTGAAGGTTAACATTAACATCGAAGAGCGCGAGGGCCAGAACAACATCCGTATCGAGGGTCCCAAGGAGGGAGTACGGCAGGCGCAGCTTGAATTACAAGAAAAAATCGACAAACTGGAAAACGAAAAATCGAAGGATGTGATCATCGACCGCCGTCTCCATCGTTCTATTATCGGAGCTAAGGGCGAGAAGATTCGCGAGGTGAAGGACCGCTACCGCCAGGTTACAATCACGATACCTACGCCCCAGGAGAATACCGATATTGTGAAGCTGCGCGGACCCAAGGAGGATGTGGACAAGTGTCACAAGGATCTGCTTAAGCTGGTCAAGGAGATTCAGGAATCGTCGCACATTATCGAGGTGC

>RpS3

CATTGAGTTGTACGCCGAGAAGGTGGCCGCTCGTGGCCTGTGCGCCATTGCCCAGGCTGAGTCGCTGAGGTACAAGCTCACCGGAGGACTGGCCGTCCGTCGTGCTTGCTATGGTGTGCTCCGCTACATCATGGAGTCGGGAGCCAAGGGCTGCGAGGTCGTCGTGTCCGGCAAACTGCGTGGTCAGCGTGCCAAGTCGATGAAATTCGTCGATGGCCTGATGATCCATTCGGGAGATCCGTGCAACGACTATGTCGAGACCGCCACCCGTCATGTGCTCCTCCGCCAGGGAGTGCTTGGTATCAAGGTCAAGGTCATGTTGCC

>Cnot4

CTAGCAATAGAACGAGGGCGGATCGTGGAAAAGATCGGACCACGGCTAGTGCAAAGGAGCAGAAGAAGAGCAAGGAAGCTGCTCCAGCACCTGCAGCAAGTAAACCGGCGGAGCGGGTTGAAACAAGCGAGAGTACAATAAGACAAAAGAAGGCGGAAGTAACAGAAAGCTGTGAAGATAACTTACCACAAAAGAGATTAGCGGGAACAAACGTTCAAAGATCTGTGAGCTCTTGTAGCGAAAATAGCGAAGGACACGTCTCTGAGAGTAGCTTAAGTGAGAAGAGTTTAACTGGTGATTATGTGGAGGAAAAGTGCAATAGTGTGAATTCGGAAAGCCAGCAAGAAAGTG

>eIF3-S9

CCTGGAGAAGCTGAAGTTGGTCATCAACAAGCTGTTTTCGAACTACGGAGAAATCGTCAATGTGGTCTATCCCGTCGACGAGGAGGGCAAGACCAAGGGCTACGCCTTCATGGAGTACAAGCAGGCCAGACAGGCGGAGGAAGCCGTCAAGAAGCTCAACAATCATCGCCTAGACAAAAACCACACCTTTGCCGTCAATCTCTTCACCGATTTCCAAAAGTACGAAAACATCCCCGAGAAGTGGGAGCCGCCAACCGTGCAGACCTTCAAAGTGC

>rin

AGATCCACAACCGAATCCAGCAGCTGAACTTCAACGATTGCCACGCGAAGATCAGCCAGGTTGATGCCCAGGCCACTTTGGGCAACGGTGTGGTGGTTCAGGTCACCGGGGAGCTATCCAATGATGGCCAGCCGATGCGGCGTTTTACCCAGACGTTCGTTCTGGCCGCTCAGTCGCCGAAGAAGTACTACGTGCACAACGACATCTTCCGCTATCAGGATCTCTACATCGAGGACGAGCAGGATGGCGAGTCGCGATCGGAGAACGATGAGGAGCACGAT

>barc

GGAGAATACAATCCCGCTCTGAAGCCCAAACGCAAGAAGAAGGACAAAGAGAAATTGCAAAAGATGAAGGAAAAGTTATTTGATTGGCGTCCAGATAAATTGCGTGGCGAACGGTCAAAGAATGAGAAAACCGTCATCATTAAAAACCTCTTCACCCCAGAACTCTTTGAGAAGGAAGTGGAGCTCATATTGGAGTACCAAAACAATCTGCGTGAGGAGTGCAGCAAATGCGGGATGGTCCGTAAAGTGGTTATCTATGATCGCCATCCTGATGGTGTAGCCCAGATCAACATGGCCTCGCCGGAGGAAGCTGACCTCGTCATTCAAATGATGCAGGGGCGTTATTTTGGACAGCGGCAACTAAGTGCGGAGGCCTGGGATGGCAAGACCAAATACAAAATTGAGGAATCAGCTGTCGAGGCGCATGAACGGCTTTCCAAATGGGATGAATTCTTGGCAGAAG

AAGAAACCG

>eIF3ga

GAGGTGGAGCTCGACTATGGTGGACTACCTCCGACGACGGAGACGGTGGAGAACGGACAGAAGTACGTGACGGAGTACAAGTACAACAAGGACGACAAGAAGACGAAGGTGGTGCGCACGTACAAGATATCCAAGCAGGTGGTGCCCAAGACGGTGGCCAAGCGACGCACCTGGACGAAGTTCGGCGACTCGAAGAACGACAAGCCCGGCCCCAACTCGCAGACGACCATGGTGTCCGAGGAGATCATCATGCAGTTCCTCAACTCCAAGGAGGACGAGAAGGCCAACGATCCGCTGCTAGATCCCACCAAGAATATTGCCAAGTGCCG

>RnpS1

ATTCATGTCGGTCGGCTTACCCGCAACGTTACCAAGGACCATGTGTTCGAGATATTTAGCAGCTTTGGGGATGTGAAGAATGTGGAGTTTCCCGTAGATCGTTTTCATCCTAACTTCGGACGCGGCGTGGCGTTTGTGGAATATGCCACACCCGAGGATTGTGAGTCGGCCATGAAGCATATGGATGGCGGGCAGATAGATGGCCAGGAGATTACGGTATCCCCGGTTGTCTTAGTAAAACAGAGGCCGCCCATGCGTCGTCCTTCGCCACCGATGCGCCGTCCGCAAAACAACCGCTGGCGATCCCCACCCCAGTTCAATAGGTTCAACAATCGTGGAGG

>Ref1

AACAGCGCTTGGAAGCACGATATGTACGACGGACCGAAGAGGGGTGCCGTCGGTGGAGGATCTGGACCCACCCGCCTCATCGTCGGTAACCTGGACTACGGCGTATCCAACACGGACATCAAGGAGCTCTTCAACGACTTTGGTCCGATAAAGAAGGCGGCAGTGCACTACGATCGCTCCGGTCGCTCGTTGGGCACCGCTGACGTGATTTTCGAACGTCGCGCCGACGCCTTGAAGGCCATTAAACAGTACCATGGCGTACCTTTGGACGGACGCCCTATGACCATTCAGCTGGCCGTCTCAGACGTGGCCGTGTTGACCCGTCCCGTAGCCGCCACCGATGTCAAGCGTCGCGTGGGTGGTACTGCACCAACTTCATTCAA

>tsu

CCGATGTGTTGGACATTGACAATGCGGAGGAGTTCGAGGTGGACGAGGACGGTGACCAGGGCATTGTGCGCCTGAAGGAAAAGGCGAAGCACCGCAAGGGACGCGGATTTGGAAGCGACAGTAACACCCGAGAGGCGATCCACAGCTACGAGCGTGTGCGCAACGAGGACGACGATGAGCTGGAACCTGGTCCACAAAGGTCCGTCGAGGGCTGGATACTGTTTGTCACCTCTATCCATGAGGAGGCGCAGGAGGACGAGATTCAGGAAAAGTTCTGCGATTACGGAGAAATCAAGAACATTCACCTGAACCTCGACCGGCGTACTGGGTTCTCAAAGGGATACGCTCTCG

>shep

ATGGATTCCGGGTTACATGATGACTCAGGTAGATGATCAGACTTCGTATTCTCCACAGTACATGCAGATGGCAGCTGCCCCTCCGCTGGGAGTAACCTCATACAAACCGGAGGCGGTTAACCAGGTGCAGCCCCGTGGCATCTCGATGATGGTTAGCGGTGATACGGGCGTGCCATATGGAACAATGATGCCTCAGTTGGCCACCCTGCAGATTGGCAACTCTTATATTAGTCCAACTTATCCATATTATGCACCACCACCAACTATTATACCAACAATGCCAATGACAGATTCCGAACAGGCTAGCA

>snRNP-U1-70K

TTTAAGACGAGGAACTTCAGGAAAAGGTAAAACAAAACAAAAAAGCCCACAAAATGACCCAATATCTGCCGCCGAATCTGCTGGCGCTGTTCGCGGCACGGGAGCCCATCCCGTTCATGCCGCCGGTGGACAAGCTGCCGCACGAGAAGAAGTCTCGCGGCTACCTGGGAGTGGCCAAGTTCATGGCCGATTTCGAGGATCCCAAGGACACGCCGCTGCCGAAAACGGTGGAAACGCGTCAGGAGCGGCTGGAGCGACGCCGGCGCGAGAAGGCCGAGCAAGTGGCCTACAAGCTGGAGCGTGAGATAGCGCTGTGGGACCCCACAGAGATCAAAAATGCCACGGAGGACCCGTTTCGCACGCTGTTCATTGCACGCATCAACTACGACACGTCCGAGTCGAAGCTGCGGCGTGAGTTCGAGTTCTACGGGCCCATCAAGAAGATCGTCCTGATCCACGACCAGGAATCAGGTAAACCCAAGGGCTACGCCTTCATCGAGTACGAGCA

>Caper

ACACGCAGGCTGAGAAGAATCGTCTCCAGAATGCAGCGCCGGCATTCCAACCGAAGAGTCACACGGGTCCCATGCGCCTCTACGTGGGATCACTGCACTTCAACATTACCGAGGACATGCTGCGGGGCATATTCGAGCCCTTTGGCAAGATCGATGCCATTCAACTGATCATGGATACGGAGACGGGCCGATCCAAGGGCTACGGCTTTATCACGTACCACAATGCTGACGATGCCAAAAAGGCTCTGGAACAGCTGAACGGCTTTGAACTGGCCGGTCGGCTCATGAAAGTGGGCAATGTGACGGAGCGACTGGACATGAATACCACCTCGCTGGACAC

>nonA-l

AACTGATGACGACCTACGGGAGATGTTCAAGCCATATGGCGAGATCGGCGATATATTCTCGAACCCGGAGAAGAACTTTACATTCCTGAGGCTAGACTACTACCAAAATGCTGAGAAGGCCAAACGCGCTTTAGATGGCTCCTTGCGCAAGGGACGAGTGCTGCGTGTCCGCTTTGCGCCCAACGCCATTGTGCGTGTGACTAATCTCAACCAGTTCGTGTCCAACGAGCTGCTGCACCAGTCCTTTGAGATCTTTGGACCCATCGAGCGCGCCGTTATCTGCGTAGACGATCGCGGTAAGCATACCGGCGAAGGCATTGTTGAGTTCGCCAAGAAGTCCTCGGCCAGCGCCTGTCTGCGCCTGTGCAACGAAAAATGCTTCTTCTTGACTGCTTCATTGCGTCCGTGTCTGGTGGAACCGATGGAGGTGAACAACGACAATGACG

>Hrb27C

TGAGCACGTGACCAACGAGCGGTACATCAATCTGAATGGCAAGCAGGTCGAAATCAAGAAGGCCGAGCCTCGTGATGGATCTGGCGGCCAAAACTCCAACAACAGTACCGTGGGAGGCGCCTATGGCAAGCTTGGTAACGAGTGCAGCCACTGGGGACCGCACCATGCTCCCATCAACATGATGCAGGGCCAGAATGGCCAGATGGGTGGACCGCCGCTGAATATGCCCATTGGAGCGCCGAATATGATGCCTGGCTATCAGGGTTGGGGCACCTCGCCGCAGCAGCAACAATACGGCTACGGCAACAGTGGCCCAGGATCGTACCAGGGATGGGGAGCTCCACCAGGACCCCAGGGACCACCACCGCAGTGGTCGAACTACGCTGGACCTCAGCAGACGCAGGGCTACGGCGGATACGACATGTATAACTCGACGTCGACCGGAGCTCCTTCGGGACCATCGGGCGGCGGCAGCTGGAACTCGTGGAACATGCCACCTA

>Sxl

TAATCTCTGCGGATTGTCGCTGGGCAGCGGTGGTAGTGATGATCTCATGAACGATCCTCGGGCAAGCAACACCAACCTGATTGTCAACTACTTGCCCCAGGACATGACCGATCGCGAGCTGTACGCCCTATTCAGAGCCATTGGACCCATCAACACGTGCAGAATCATGCGAGACTATAAGACTGGCTACAGTTTTGGTTATGCTTTCGTGGACTTCACATCGGAAATGGACTCGCAGCGTGCTATTAAAGTGCTGAATGGCATCACAGTGCGCAACAAGCGGCTTAAGGTTTCCTATGCACGTCCCGGCGGAGAATCGATCAAGGACACCAATCTGTATGTGACCAATCTGCCGCGTACCATAACCGACGATCAGCTGGACACGATCTTCGGCAAGTACGGTTCCATTGTGCAGA

>Rox8

CCGGTGTAAAGGGAAGTCAACGCCACACCTTCGAGGAAGTGTATAACCAGTCGAGCCCCACCAACACCACCGTATACTGTGGCGGATTCCCGCCGAATGTCATCAGTGACGACCTGATGCACAAGCACTTCGTCCAGTTTGGTCCCATCCAGGACGTGCGGGTCTTCAAGGACAAGGGCTTCTCGTTCATCAAGTTTGTTACCAAGGAGGCAGCCGCCCACGCCATCGAGCACACGCACAACAGCGAGGTACATGGAAACCTGGTAAAGTGCTTCTGGGGCAAAGAGAACGGAGGCGATAACTCGGCCAATAACCTCAATGCCGCCGCTGCCGCGGCAGCAGCCTCTGCCAATGTTGCCGCCGTTGCGGCAGCCAATGCTGCGGTTGCCGCTGGAGCGGGTATGCCCGGTCAGATGATGACGCAGCAACAG

>bol

CTGATCTAACCCGCGTCTTCAGCGCCTATGGCACGGTAAAGAGCACCAAAATCATCGTGGATCGAGCAGGTGTGAGCAAGGGCTACGGATTCGTCACCTTCGAGACGGAGCAGGAGGCGCAAAGACTGCAAGCGGATGGTGAATGCGTGGTACTAAGAGATCGGAAGCTGAACATTGCACCGGCCATCAAAAAGCAGCCCAATCCTCTGCAGTCAATTGTGGCCACAAACGGAGCCGTCTACTATACCACCACGCCGCCGGCACCGATCAGCAATATACCCATGGATCAGTTCGCAGCCGCTGTATATCCGCCAGCCGCTGGAGTGCCAGCCATCTACCCACCTTCAGCCATGCAATATCAGCCATTCTATCAGTACTACAGTGTGCCAATGAATGTACCCACCATTTGGC

>Rnp4F

CAGGAGGAGGAGCACAAGTCGGAGGAGCTGCGCCAACGATCGCGCCCAACCTGGCCACCGTCGTCCGCCGGCGGGGATATGACCACCATTGAGTTGATCTCATCGGACGACGAGCCGTCAGTGGAGGAGACTGAGGGAGGCAATGCCGCTGGCCGTGGCAGAGCGCGCAATGATTCCAGCAGCAGTAGCGATGATGTGGGCGTGATCGAAGGCTCGGAATTGGAATCGAACAGTGAGGTGTCCAGTGACAGTGACAGTGATAGCGACAACGCTGGCGGCGGAAATCAGCTAGAGCGCTCGTATCAGGAGCTGAATGCGTTGCCCAGCAAAAAGTTTGCCCAAATGGTCTCGCTCATTGGAATCGCATTCAAA

>elav

TCGGGATCGCAAAATGGCAGCAACGGCAGCACGGAGACGCGCACAAACCTTATTGTCAACTACTTGCCGCAAACAATGACCGAAGACGAGATCCGTTCGCTCTTCTCCAGCGTCGGCGAGATTGAGTCGGTGAAGCTGATACGCGACAAGTCGCAGGTCTACATCGATCCTCTCAATCCGCAGGCGCCCAGCAAGGGCCAAAGTCTGGGCTACGGCTTTGTTAACTATGTCCGGCCGCAAGATGCCGAGCAGGCTGTTAATGTTCTAAACGGCCTGCGACTGCAGAACAAAACCATAAAGGTGTCGTTTGCCCGCCCGTCGTCCGATGCCATTAAAGGCGCCAACCTTTATGTGTCGGGGCTGCCAAAGACGATGACCCAGCAGGAACTGGAGGCCATCTTCGCACCATTCGGAGCAATAATCACATCGCGCATTCTGCAGAACGCTGGCAACGATACGCAGACGAAAG

>pea

ATGGACGAGCTGCAGAAGTTGGAGTACCTTTCGCTGGTCTCGAAGATTTGCACTGAGCTAGACAACCACTTGGGCATCAACGACAAGGACCTGGCCGAGTTTATCATCGATTTAGAAAACAAAAATCGCACATATGACACATTTCGCAAGGCTTTGCTGGATAATGGCGCCGAATTCCCAGACTCCCTGGTCCAGAACCTGCAGCGCATCATTAATCTTATGCGCCCCAGCAGACCTGGCGGCGCTAGCCAGGAGAAAACTGTCGGCGACAAGAAGGAAGACAAGAAATCGCAACTTTTGAAAATGTTTCCCGGCCTCGCTTTGCCCAATGACACCTACA

>CG1646

AATATAATCCGGGCAGTCCCACATCTGAGAGCAACGACGCACAGCCCTCAGAGAAAAAACTCAAGGTCGAAGAATCGGAGCCCAAGGAGAAAAAGAAGGAAAAGGAGCGCGATAAAGATAAGGAGAAGGATAAGGACAAAGATAATAATAAGGATAAGGAAAAGGAGCGAAAGAAGCTGCCGGACCTAGATAAGTACTGGAGAGCTGTCAAAGAAGACTCCACCGACTTCACCGGCTGGACGTACTTGCTGCAATATGTTGACAATGAGTCTGATGCGGAGGCGGCGCGCGAGGCCTACGACACATTCCTGTCCCACTATCCTTACTGCTACGGATATTGGCGCAAGTATGCCGACTACGAGAAGCGCAAGGGCATCAAGGCAAACTGCTATAAGGTGTTTGAGCGCGGACTGGAGGCGATTCCGCTGTCCGTGGATCTGTGGATCCACTACCTAATGCACGTTAAGTCCAATCACGGAGATGATGA

>CG6227

GTGCCCAACCACTACGAGGACTATGTTCACAGATGTGGTCGCACCGGTCGAGCGGGCAAAAAGGGCAGCGCCTACACGTTTATCACACCGGAGCAATCGCGCTATGCCGGCGACATTATCCGCGCCATGGACCTATCAGGCACACTGATTCCCGCCGAGCTGCAGGCACTGTGGACGGAGTATAAGGCGCTCCAGGAGGCCGAGGGCAAGACGGTGCACACGGGCGGCGGCTTTAGCGGCAAGGGCTTCAAGTTCGACGAGCAGGAGTTCAATGCCGCCAAGGAGAGCAAGAAGCTGCAGAAGGCGGCCTTGGGACTGGCCGATTCCGATGATGAGGAGGA

>CG6841

ATGCCCTCCAAATATTTCCCTCGAAGAAAAGCATCTGGTTGCGAGCCGCCTACTTTGAAAAGAACCATGGCACCCGCGAATCTTTGGAGGCCCTGTTGCAGCGAGCCGTGGCTCATTGTCCTAAATCGGAGATTCTCTGGCTGATGGGGGCCAAATCCAAATGGATGGCTGGAGACGTTCCAGCCGCGAGAGGCATTTTGTCCTTGGCTTTCCAGGCCAATCCCAATTCCGAGGACATTTGGTTGGCTGCCGTTAAGTTGGAATCAGAGAACTCGGAATATGAGCGGGCGAGACGCTTGTTAGCCAAGGCTAGAGGATCGGCACCGACACCAAGGGTGATGATGAAATCAGCTCGCCTGGAATGGGCTTTGGAAAAGTTCGACGAAGCT

>Rm62

CATCTACGACACCAGCGAGAGCCCCGGCAAGATTATCATATTCGTGGAGACAAAGCGACGCGTGGACAACCTGGTGCGCTTCATCCGCAGCTTCGGAGTCCGTTGTGGAGCTATTCACGGTGACAAGTCGCAATCAGAACGAGACTTTGTGCTCCGTGAGTTCCGCTCGGGCAAGTCCAACATTCTGGTGGCCACCGATGTGGCGGCCCGTGGACTAGACGTGGACGGCATCAAGTATGTCATCAACTTTGACTACCCGCAAAACAGCGAGGACTACATCCATCGCATCGGTCGCACAGGACGATCCAACACAAAGGGCACCTCTTTCGCCTTCTTCACCAAG

>hay

GATCACGGAAATCGACCACTTTGGGTTGCGCCCAATGGTCACGTCTTCCTGGAATCATTCTCGCCCGTCTATAAGCATGCCCACGATTTTCTTATCGCCATTTCGGAGCCCGTCTGCCGACCCGAACACATTCACGAGTACAAACTTACCGCATACAGTTTATATGCCGCCGTTTCGGTGGGACTGCAAACCCATGACATTGTGGAATACTTGAAGAGATTGAGCAAGACCAGCATTCCCGAAGGCATCCTTGAGTTTATACGACTCTGCACCCTATCCTATGGCAAGGTCAAGCTGGTCTTGAAGC

>qkr54B

AACTGCTGGAAGGCGAGATAGAAAAGGTCCAGACCACAGGAAGGATTCCTTCCAGAGAGCAAAAGTATGCCGATATCTATAGAGAGAAGCCGCTGCGGATCTCGCAACGTGTTTTAGTTCCCATTAGAGAACATCCCAAGTTCAACTTCGTTGGAAAACTGCTGGGGCCCAAGGGCAACTCCCTTCGCCGCCTTCAGGAGGAGACCCTTTGCAAGATGACCGTCCTGGGCCGCAACTCTATGCGCGATCGAGTCAAAGAAGAGGAATTGCGCAGCTCCAAGGATCCCAAGTACGCTCACCTCAACAGCGATCTGCATGT

>qkr58E-3

AACGACGAGGTTTCACACGAACAGCTGCGCGAGCTGATGGAAATGGATCCCGAGTCAGCCAAAAACATTCACGGACCGAATCTGGAGGCCTACAGATCTGTCTTCGACAAGAAGTTTGGAGGCAACAGCAATGGGGCTCCCAAATACATCAACCTGATTAAGAGAGCTGCGGAAAATCCGCCCGAAGTCGACGATGTGGAGGAGGTGGCCTATGAGTATGAACATCGTATGCCCCCCAAGCGTCCGCCTACGGGCTATGAGTACAGCAAACCACGTCCATCAATAATACCGACAAACGCAGCGGCATATAAACGTCCATATCCGACTGACATG

**RNA-seq**

RNA-seq libraries were prepared from 10 µg of total RNA from each sample using the Illumina mRNA-seq library preparation kits as described by the manufacturer. Libraries were sequenced on an Illumina GAIIx using single reads of 75 or 76 bp in length. Each knockdown was performed in biological duplicate. After quality control analysis of the correlation of junction and exon read counts, duplicate samples were combined.

### RNA-seq alignment strategy

RNA-seq reads were aligned with Bowtie against a reference sequence consisting of the genome, annotated splice junctions, and unannoated splice junctions. All RNA-seq reads were trimmed to 75 base pairs for consistency. To ensure a 6 bp overhang, splice junction sequences were formed by joining sequences 69 bp of exon on each side of the splice junction.

Novel splice junctions included all novel combinations of exon-exon junctions within the same gene and different genes, with splice sites within 2 kb of each other. No length restrictions were made for novel junctions within the same gene. Additional novel junctions were derived from an annotated splice site and an unannotated splice site (GT or AG dinucleotide) within 2 kb away.

To remove potential false positive novel junctions, each unannotated splice junction was given a Shannon-entropy score as previously described . Any novel junction with an entropy score ≥ 3 in one of the 57 samples (56 RNAi samples + 1 untreated) was used for further splicing analysis.

### Identifying potential RNAi off-target effects

To determine instances in which the dsRNAs used to deplete the target transcripts encoding RNA binding proteins may have had unintended targets, we first constructed a Bowtie index of the MDv1 transcriptome annotation . We then generated a set of all possible 20 nt dsRNA fragments by sliding a 20 nt window over the dsRNA sequences and then used Bowtie (with options --all -y -v 2) to align the dsRNA fragments to the transcriptome index. There appear to be potential off-target effects for other RNA binding proteins in two cases. First, we identified multiple dsRNA fragments from the *msi* dsRNA that aligned to the *Hrb87F (hrp36)* mRNA and observed significant decrease in *Hrb87F* gene expression in the *msi* RNAi sample (Supplementary Figure 4). We therefore excluded the *msi* RNAi experiments from our analysis, but the raw data is still available from GEO and the modENCODE data repository. Similarly, there are 70 20 nt fragments from the *Hrb87F (hrp36)* dsRNA that align to the *Hrb98DE (hrp38)* gene and there is a ~2.2-fold change in the levels of *Hrb98DE (hrp38)* in the *Hrb87F (hrp36)* RNAi sample. As the impact on the potential off-target is less than that in the *msi* case, we have kept this sample in the analysis, though it is possible that off-target effects may account for some of the effects observed in the *Hrb87F (hrp36)* RNAi samples.

**Supplemental References**

Boyle AP, Araya CL, Brdlik C, Cayting P, Cheng C, Cheng Y, Gardner K, Hillier L, Janette J, Jiang L et al. 2013. Comparative analysis of regulatory information and circuits across diverse species. *Nature*: Submitted.

Brooks AN, Hansen KD, Hundal A, Dudoit S, Meyerson M, Brenner SE. 2013. JuncBASE: a junction-based analysis of splicing events from RNA-seq data. Submitted.

Brooks AN, Yang L, Duff MO, Hansen KD, Park JW, Dudoit S, Brenner SE, Graveley BR. 2011. Conservation of an RNA regulatory map between Drosophila and mammals. *Genome Res* **21**(2): 193-202.

Cherbas L, Willingham A, Zhang D, Yang L, Zou Y, Eads BD, Carlson JW, Landolin JM, Kapranov P, Dumais J et al. 2011. The transcriptional diversity of 25 Drosophila cell lines. *Genome Res* **21**(2): 301-314.

Finn RD, Mistry J, Tate J, Coggill P, Heger A, Pollington JE, Gavin OL, Gunasekaran P, Ceric G, Forslund K et al. 2010. The Pfam protein families database. *Nucleic Acids Res* **38**(Database issue): D211-222.

Gattiker A, Gasteiger E, Bairoch A. 2002. ScanProsite: a reference implementation of a PROSITE scanning tool. *Applied bioinformatics* **1**(2): 107-108.

Graveley BR, Brooks AN, Carlson JW, Duff MO, Landolin JM, Yang L, Artieri CG, van Baren MJ, Boley N, Booth BW et al. 2011. The developmental transcriptome of Drosophila melanogaster. *Nature* **471**: 473-479.

Hulo N, Bairoch A, Bulliard V, Cerutti L, De Castro E, Langendijk-Genevaux PS, Pagni M, Sigrist CJ. 2006. The PROSITE database. *Nucleic Acids Res* **34**(Database issue): D227-230.

Jain E, Bairoch A, Duvaud S, Phan I, Redaschi N, Suzek BE, Martin MJ, McGarvey P, Gasteiger E. 2009. Infrastructure for the life sciences: design and implementation of the UniProt website. *BMC Bioinformatics* **10**: 136.

Langmead B, Trapnell C, Pop M, Salzberg SL. 2009. Ultrafast and memory-efficient alignment of short DNA sequences to the human genome. *Genome Biol* **10**(3): R25.

Letunic I, Doerks T, Bork P. 2009. SMART 6: recent updates and new developments. *Nucleic Acids Res* **37**(Database issue): D229-232.

Negre N, Brown CD, Ma L, Bristow CA, Miller SW, Wagner U, Kheradpour P, Eaton ML, Loriaux P, Sealfon R et al. 2011. A cis-regulatory map of the Drosophila genome. *Nature* **471**(7339): 527-531.

Park JW, Graveley BR. 2005. Use of RNA interference to dissect the roles of trans-acting factors in alternative pre-mRNA splicing. *Methods* **37**(4): 341-344.

Park JW, Parisky K, Celotto AM, Reenan RA, Graveley BR. 2004. Identification of alternative splicing regulators by RNA interference in Drosophila. *Proc Natl Acad Sci USA* **101**(45): 15974-15979.

Trapnell C, Williams BA, Pertea G, Mortazavi A, Kwan G, van Baren MJ, Salzberg SL, Wold BJ, Pachter L. 2010. Transcript assembly and quantification by RNA-seq reveals unannotated transcripts and isoform switching during cell differentiation. *Nat Biotechnol* **28**(5): 511-515.
